# Recent Advances and Strategies toward Polysulfides Shuttle Inhibition for High‐Performance Li–S Batteries

**DOI:** 10.1002/advs.202106004

**Published:** 2022-03-01

**Authors:** Youzhang Huang, Liang Lin, Chengkun Zhang, Lie Liu, Yikai Li, Zhensong Qiao, Jie Lin, Qiulong Wei, Laisen Wang, Qingshui Xie, Dong‐Liang Peng

**Affiliations:** ^1^ State Key Lab for Physical Chemistry of Solid Surfaces Fujian Key Laboratory of Materials Genome Collaborative Innovation Center of Chemistry for Energy Materials College of Materials Xiamen University Xiamen 361005 P. R. China; ^2^ Shenzhen Research Institute of Xiamen University Shenzhen 518000 P. R. China

**Keywords:** electrolyte systems, functional separators, lithium anode, shuttle effect, sulfur hosts

## Abstract

Lithium–sulfur (Li–S) batteries are regarded as the most promising next‐generation energy storage systems due to their high energy density and cost‐effectiveness. However, their practical applications are seriously hindered by several inevitable drawbacks, especially the shuttle effects of soluble lithium polysulfides (LiPSs) which lead to rapid capacity decay and short cycling lifespan. This review specifically concentrates on the shuttle path of LiPSs and their interaction with the corresponding cell components along the moving way, systematically retrospect the recent advances and strategies toward polysulfides diffusion suppression. Overall, the strategies for the shuttle effect inhibition can be classified into four parts, including capturing the LiPSs in the sulfur cathode, reducing the dissolution in electrolytes, blocking the shuttle channels by functional separators, and preventing the chemical reaction between LiPSs and Li metal anode. Herein, the fundamental aspect of Li–S batteries is introduced first to give an in‐deep understanding of the generation and shuttle effect of LiPSs. Then, the corresponding strategies toward LiPSs shuttle inhibition along the diffusion path are discussed step by step. Finally, general conclusions and perspectives for future research on shuttle issues and practical application of Li–S batteries are proposed.

## Graphical abstract

This review summarizes the recent advances and strategies to suppress the shuttle effect of lithium polysulfides (LiPSs) in lithium–sulfur batteries. These strategies are composed of using the modified sulfur hosts to immobilize LiPSs, electrolyte systems to alleviate shuttle behavior, functional separator to intercept LiPSs, and anode surface engineering to avoid the chemical reaction between LiPSs and Li.

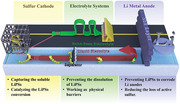

## 1 Introduction

Increasing demand of electric storage systems has considerably promoted the development of high‐energy‐density batteries, rechargeable lithium‐ion batteries have penetrated into every aspect of the modern society.^[^ ^1^ ^]^ However, current commercialized lithium (Li) ion batteries based on conventional insertion cathode and graphite anode materials have almost approached their theoretical energy density, and cannot meet the requirements for higher energy density energy storage devices, such as electric vehicles, drones, etc.^[^ ^2^ ^]^ Therefore, it is urgently needed to pursue other new battery systems with high energy density. Of the explored candidates, lithium–sulfur (Li–S) batteries have been widely studied as one of the most promising next‐generation energy storage systems due to their high theoretical specific capacity of 1675 mAh g^‐1^ and overwhelming energy density of 2600 Wh kg^‐1^, which is much higher than that of the traditional insertion‐type Li‐ion battery systems.^[^ ^3^ ^]^ What is more, due to the abundance reserves, low cost, and environmental friendliness of sulfur, the practical application of Li–S batteries has gained more competitive advantages.^[^ ^4^ ^]^ Despite all these merits, Li–S batteries have undergone a tortuous developing road and multiple intrinsic drawbacks need to be addressed before their practical application. These obstacles include: 1) the solid sulfur and Li_2_S are inherent insulative, leading to the low utilization of active materials and large electrochemical polarization.^[^ ^4a^ ^]^ 2) The high volume expansion (≈80%) of solid sulfur into Li_2_S exerts heavy stress on the host structure and causes the detachment of sulfur species from the conductive substrate.^[^ ^5^ ^]^ 3) The notorious shuttle effect caused by soluble lithium polysulfides (LiPSs) results in the loss of active materials and rapid capacity decrease.^[^ ^6^ ^]^ 4) The Li dendrite growth induced by the nonuniformity Li deposition and the passivation layer formed by chemical reactions between active LiPSs and fresh Li would lead to the rapid decay of batteries and potential safety hazard.

**Figure 1:**
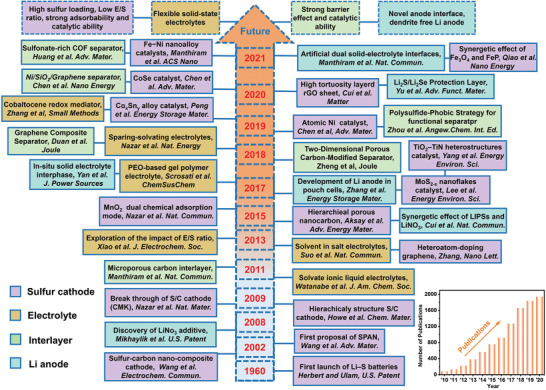
Overview of the development of Li–S batteries from the first proposal to the present. Highlighting seminal advancements in the sulfur cathode, electrolyte, interlayer, and Li anode. Inset in the lower right corner is the number of publications in recent years with the topic keywords of “Li–S battery” and “lithium–sulfur battery” in the Web of Science database, dated December 1st, 2021.

Tremendous efforts have been made to address the aforementioned challenges of Li–S batteries from the beginning to the near. **Figure** **1** details major milestones in the development of Li–S batteries to date. The prototype of Li–S batteries was proposed first by Herbet and Ulam in the 1960s and early researches mainly focused on how to make the battery run reversibly. After a period of silence, until the 21st century, significant breakthroughs in cathode design have significantly improved the cycling stability of Li–S batteries and set off a research boom in Li–S batteries again (purple). In 2002, the composite S/porous carbon cathode was demonstrated by Wang et al. with favorable sulfur utilization and improved cyclability.^[^ ^7^ ^]^ More importantly, Nazar and co‐workers proposed a pioneering composite S/mesoporous carbon (S/C, carbon: CMK‐3) cathode in 2009, a high specific capacity of 1320 mAh g^−1^ and long cycle lifetimes were achieved, initiating the booming development of cathode for Li–S batteries.^[^ ^4^, ^8^ ^]^ Targeting the same challenges, the development of novel electrolyte design (brown) and separator modification (light green) can also effectively improve cell performance.^[^ ^9^ ^]^ In addition, the further success in the engineering of stable anode solid electrolyte interphases (SEI), such as the discovery of LiNO_3_ additive for liquid electrolyte, has boosted the Coulombic efficiency and brought Li–S batteries ever closer to practical applications. As summarized above, it is not difficult to find that the development history of Li–S batteries is closely accompanied by the development of polysulfides shuttle suppression, and the cycle performance of batteries is too difficult to satisfy the practical demand as long as the shuttle effect of polysulfides exists. As the battery cycles, such a shuttle behavior of LiPSs will cause low Coulombic efficiency, rapid capacity decay, and lithium metal corrosion. More seriously, all these issues will turn more severe when the sulfur loading increases, especially in the pouch cells at the practical application level.^[^ ^10^ ^]^

**Figure 2:**
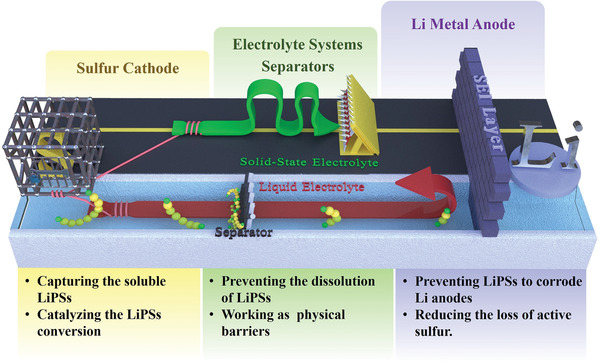
Schematic illustrations of the strategies and operation mechanisms of the modified sulfur host, electrolyte systems, functional separators, and anode surface engineering for the inhibition of LiPSs shuttle.

In this regard, a comprehensive, thorough, and in‐depth understanding of the shuttle effect is very important and meaningful for the design of high‐performance Li–S batteries with high specific capacity and stable cyclic ability. From the point view of LiPSs, when solid sulfur accepts Li^+^ ions from the liquid electrolyte, it will convert into soluble long‐chain LiPSs. If long‐chain LiPSs cannot be well captured by host materials to undergo the subsequent redox reaction, they will diffuse into the electrolyte. And then, the viscosity of the electrolyte will continue to increase as the LiPSs dissolve, which greatly increases the transfer resistance of Li^+^ ions. Driven by the concentration gradient, the soluble long‐chain LiPSs penetrate the separator to the anode side, and the long‐chain LiPSs will react with the Li metal to generate short‐chain LiPSs, or form a solid Li_2_S and Li_2_S_2_ layer cover on the surface of the Li metal anode, blocking the passage of Li^+^ ions. As a consequence, we can see that the soluble LiPSs exert a certain negative effect on the corresponding components along the shuttle path. In this comprehensive review, as illustrated in **Figure** **2**, we specifically concentrate on the shuttle path of LiPSs and their impact and harm on these components, systematically summarize and discuss the recent advances of the cathode, electrolyte, separator, and anode in inhibiting the shuttle effect. The fundamental aspects of the Li–S batteries are presented at first to give an in‐deep understanding of the ins and outs of the shuttle effect. Next, the corresponding strategies of shuttle effect inhibition are classified and discussed. Finally, conclusions and perspectives insight for the future research emphasis are provided. By reviewing the rational design and engineering of these components in inhibiting the shuttle effect, we intend to provide a profound understanding and inspiration on the shuttle effect and further provide guidance for the future design of high‐performance Li–S batteries.

## 2 Electrochemical Principles and Shuttle Effect of Li–S Batteries

**Figure 3:**
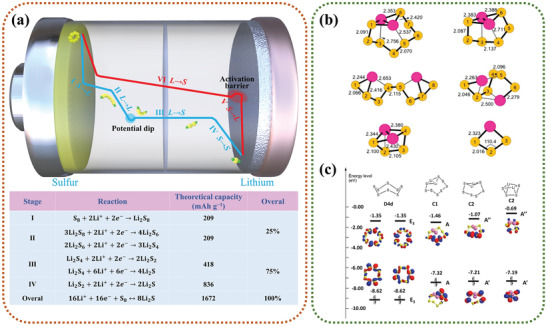
a) Schematic illustration and operating principles of Li–S batteries, the inset is the discharge‐charge curves. b) Stable optimized structures and symmetries of neutral Li*_x_* S*_n_* species. c) LUMO and HOMO energy levels calculated for Li*_x_* S*_n_* species. Reproduced with permission.^[^ ^11^ ^]^ Copyright 2015, American Chemical Society.

The typical Li–S battery mainly encompasses sulfur cathode, lithium anode, organic electrolyte, and separator. Inset of **Figure** **3**a shows a typical charge/discharge profile, wherein two obvious discharge plateaus are observed, corresponding to a solid (S_8_)→liquid (Li_2_S*_n_*)→solid (Li_2_S_2_/Li_2_S) processes. In turn, the long charge plateau indicates that Li_2_S is decomposed into Li and sulfur in the subsequent charge process reversibly. The overall electrochemical reaction is described as:16 Li^+^ + 16 *e* ^−^ + S_8_↔8 Li_2_S.

As demonstrated in Figure 3a, although Li_2_S is the final product of discharge reaction, the redox reactions between sulfur and Li_2_S include several steps, which undergo complex composition and structure changes. Four stages can be divided during the discharge process. During stage I, solid S_8_ reacts with the migrated Li^+^ ions and electrons to form soluble long‐chain Li_2_S_8_, corresponding to a solid–liquid conversion reaction, wherein a small sloping plateau emerges at the voltage of approximately 2.4 V. Afterward, in stage II at 2.4–2.2 V, the valence of S decreases, and long‐chain Li_2_S_8_ continues to reduce to Li_2_S_6_ and Li_2_S_4_ through liquid–liquid single‐phase reactions. At the same time, it is also accompanied by the disproportionation reactions to generate long‐chain mesophase polysulfides (Li_2_S*_x_*, *x* = 4–8) due to the highly reactive polysulfides and dynamic chemical equilibrium. Dong and co‐workers reported that the energy level of the lowest unoccupied molecular orbital (LUMO) of polysulfides Li_2_S_n_ follows the order: Li_2_S_8_ (−1.46 eV) < S_8_ (−1.35 eV) < Li_2_S_6_ (−1.07 eV) < Li_2_S_4_ (0.69 eV), a lower value means a higher electronic capability (Figure 3b,c).^[^ ^11^ ^]^ For instance, S_8_ molecules can react with Li_2_S_4_ to form Li_2_S_6_ (S_8_ + Li_2_S_4_ → Li_2_S_6_). Overall, stages I and II contribute to one‐fourth of the overall theoretical specific capacity (418 mAh g^−1^), corresponding to the acceptance of 0.5 electrons per sulfur atom.

It is worth noting that the long‐chain LiPSs (Li_2_S*_x_*, 4 ≤ *x* ≤ 8) formed during such stages are easily dissolved into the easter‐based electrolytes. The good mobility of LiPSs allows themselves to dissolve into the electrolyte irreversibly and diffuse to the anode because of the concentration gradient, followed by being chemically reduced (instead of electrochemically) by Li metal to form short‐chain LiPSs or even solid Li_2_S_2_ and Li_2_S cover on the anode. Subsequently, short‐chain LiPSs will also diffuse back to the cathode and produce long ones in the following charging process. Thus, the roundtrip transport of LiPSs is the notorious “shuttle effect.” Such polysulfides shuttle phenomenon brings a series of adverse consequences for the operation of Li–S batteries. The most intuitive result is the loss of active sulfur. When the soluble polysulfides lost electrical contact with the current collector, they would not participate in subsequent electrochemical reactions, leading to the severe sulfur loss. Additionally, the Li_2_S_2_/Li_2_S passivation layer accumulated on the Li anode surface is insoluble and cannot be oxidized into long‐chain LiPSs and S_8_ again, which would not only cause the irreversible loss of active materials but also retard the diffusion and transportation of Li^+^ ions due to their poor electrical conductivity, finally leading to rapid capacity decay and limited cycle life. Furthermore, as the increase of viscosity of electrolytes due to the dissolution of LiPSs, the charge transfer resistance increases. An obvious dip‐in voltage profile at the start of the transition is formed and corresponds to the nucleation barrier of solid Li_2_S_2_/Li_2_S, and the decomposition process also needs to overcome the additional activation energy.

With the discharge process proceeding, soluble long‐chain LiPSs is further reduced to insoluble Li_2_S_2_ or Li_2_S during stage III, which is a slow liquid–solid two‐phase reaction. Reduction reactions take place simultaneously and compete with each other, resulting in a long lower voltage plateau at about 2.1 V. In the end, the last ramp stage relates to the further reduction from Li_2_S_2_ to Li_2_S. And this solid–solid conversion reaction always suffers from large polarization and slow kinetics owing to the two poorly conductive solid phases. As a whole, most of the capacity contribution comes from the latter two steps, which contributes to the other three fourth of the specific capacity of about 1254 mAh g^−1^. In the subsequent charge process, the solid Li_2_S and Li_2_S_2_ species are reversibly converted into various soluble LiPSs and then further oxidized to solid S_8_, corresponding to a long single charge plateau at about 2.4 V. Similarly, such charge process also suffers from slow kinetics as the converse transition needs to overcome additional activation energy due to the aggregation of Li_2_S product.^[^ ^12^ ^]^

In general, the operation of Li–S batteries involves multistep complex solid–liquid–solid transition accompanied by the shuttle effect. And only when the “shuttle effect” is restrained effectively, it is possible for Li–S batteries to obtain a high energy density and stable cycle ability for practical application. In the past decades, inspiring progress has been achieved in alleviating the shuttle effect in Li–S batteries. Starting from the self‐movement path of soluble LiPSs, strategies include the rational design of cathode materials, functional separators, new electrolyte systems, and anode protection, which have been studied intensively.^[^ ^13^ ^]^ Insights gleaned from numerous works, the critical issues and coping strategies of the shuttle effect are systematically summarized and shown in **Table** **1**.

**Table 1:** Effects of intermediate LiPSs on cell components and the corresponding strategies for shuttle effect inhibition along the shuttle process in Li–S batteries

Diffusion pathway	Critical issues	Strategies	Concrete methods
Sulfur cathode	Easy detachment of LiPSs from the sulfur hosts and slow kinetics of Li–S chemistry reaction	Physical confinement of LiPSs	✓ Preventing the formation of long‐chain LiPSs by microporous structure ✓ Impeding LiPSs diffusion by various dimensions of carbon‐based hosts
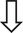		Chemical interaction towards LiPSs	✓ Adsorbing LiPSs by polar–polar interaction ✓ Adsorbing LiPSs by Lewis acid–base interaction ✓ Electrocatalysis promotes long‐chain LiPSs conversion
Electrolyte systems	Dissolution of long‐chain LiPSs into electrolyte	Tailoring the composition of liquid electrolytes	✓ Reducing the solubility of soluble LiPSs ✓ Changing the reaction pathway ✓ Regulating the electrolytes concentration
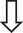		Using the solid‐state electrolytes	✓ Preventing the dissolution and shuttle of LiPSs by utilizing the inorganic solid electrolytes, solid polymer electrolytes, and composite solid electrolytes
Separators	LiPSs permeate through the separators easily and reach the anode surface	Physical shielding effect	✓ Working as physical barriers to inhibit LiPSs shuttle ✓ Adsorbing the dissociative LiPSs physically ✓ Pushing the LiPSs to cathode side by electrostatic repulsion
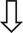		Chemical trapping effect	✓ Adsorbing LiPSs by polar–polar interaction and Lewis acid–base interaction ✓ Electrocatalysis promotes the adsorbed LiPSs conversion
Li anode	Chemical reactions between Li metal and LiPSs to corrode the Li anode	In‐situ SEI Layer	✓ Preventing the corrosion of LiPSs to Li anodes by nitrate‐containing, sulfide‐containing, halide‐containing in situ SEI layers
		Artificial protection layer	✓ Constructing inorganic, organic, and functional protective layer to avoid the harmful interfacial reaction

## 3 Strategies for Polysulfides Shuttle Inhibition along the Shuttle Process

### 3.1 Modified Sulfur Hosts

As the most crucial component of Li–S batteries, the sulfur cathode plays an essential role in capacity releasing, energy density, and cycle life. Meanwhile, on the cathode side, preventing the soluble polysulfides diffusion is the first step to suppress the shuttle effect. If there are no effective strategies to restrain the shuttle phenomenon, the soluble long‐chain LiPSs will detach from the cathode surface instantly and cannot participate in the following electrochemical reactions. In this context, considerably significant efforts based on the rational design of sulfur cathode have been made in the past few decades.

Researches on cathode modification mainly focus on the following aspects. At the very beginning, the encapsulation of sulfur molecules into the pores of various carbon hosts is considered to be an efficient way, which can physically impede the diffusion pathway to alleviate the shuttle phenomenon. Unfortunately, physical adsorption of nonpolar carbon hosts can only offer a weak interaction toward the nonpolar S_8_ via van der Waals force. And polar LiPSs will inevitably dissolve into the electrolyte. Afterward, to enhance the interaction force, chemical interaction by introducing the heteroatoms doping or polar substances has drawn tremendous interest. Heteroatoms can enhance the polarity of carbon matrices to obtain a strong interaction with LiPSs, and the combination of polar substances such as metal compounds or functionalized polymers can provide abundant adsorption active sites. However, the dissolution and diffusion of LiPSs cannot be eliminated fundamentally through only the chemical adsorption since the active sites will be saturated in a massive supply of LiPSs. Based on the sluggish multistep conversion mechanism, the rapid reduction of soluble LiPSs would be the most efficient way. Inspired by the traditional catalytic mechanism, catalysis concepts were introduced to investigate the electrocatalytic effects in Li–S batteries several years ago, and various active hosts with electrocatalytic activity have been successfully designed to enhance the conversion reaction kinetics.^[^ ^14^ ^]^ Indeed, these novel concepts and mechanisms are put forward successively, indicating a tendency of in‐depth understanding of the electrochemical reactions on the sulfur cathode. **Table** **2** summarizes the electrochemical performances of sulfur cathodes modified by such strategies.

**Table 2:** Comparison of the electrochemical performance of Li–S Batteries fabricated with various sulfur hosts

Host materials	Sulfur loading [%]	Mass loading [mg cm^−2^]	Operation voltage [V]	Initial capacity [mAh g^‐1^]/C rate	Final capacity [mAh g^‐1^]/cycle numbers	Capacity retention/decay rate [%]	Refs.
Physisorption‐confinement							
DHPCs	70	–	1.7–2.8	746/2 C	520/500 cycles	70/0.06	^[^ ^32^ ^]^
PCMSs	70	2.0	1.8–2.7	722/4 C	673/500 cycles	93.1/0.014	^[^ ^33^ ^]^
Carbon nanofiber	75	1.0	1.7–2.6	–/–	630/150 cycles	–/–	^[^ ^18^ ^]^
BCN@HCS	70	4	1.7–2.8	845/1 C	700/500 cycles	82.8/0.034	^[^ ^34^ ^]^
Nano‐S:rGO:PAQS	70	–	1.5–2.8	1255/0.5 C	559/1200 cycles	44.5/0.046	^[^ ^35^ ^]^
C/S+BTO	60	2.4	1.5–3.0	1143/0.2 C	835/100 cycles	73/0.27	^[^ ^36^ ^]^
Vermiculite	80	2	1.4–2.8	–/0.5 C	–/550 cycles	75/0.045	^[^ ^37^ ^]^
YF_3_‐doped 1D carbon Nanofibers	80	1.02	1.8–2.8	778.2/2C	597.7/800 cycles	76.8/0.029	^[^ ^38^ ^]^
				636.5/5 C	386.6/700 cycles	60.8/0.056	
CNTs/BNFs	60	–	1.7–2.8	617/4 C	482/500 cycles	78/0.044	^[^ ^39^ ^]^
PCNF/S/BPQD	68	–	1.7–2.8	810/2 C	589/1000 cycles	73/0.027	^[^ ^40^ ^]^
Polar–polar/Lewis acid–base interactions							
Nitrogen‐doped graphene	55	3.6	1.7–2.8	968.3/0.5 C	556.8/500 cycles	57.4/0.08	^[^ ^41^ ^]^
HNPC	65		1.5–3.0	1010/0.5 C	788/400 cycles	78/0.055	^[^ ^17^ ^]^
HNCM800	80	1.5	1.7–2.8	902/0.5 C	804/1000 cycles	88/0.011	^[^ ^24^ ^]^
HCMs	78	1.5	1.8–2.7	880/2 C	533/900 cycles	74/0.04	^[^ ^26^ ^]^
NPDSCS	72.4	–	1.5–3.0	952/1 C	814/500 cycles	85.5/0.029	^[^ ^27^ ^]^
Ti_4_O_7_	70	1.5–1.8	1.8–3.0	850/2 C	595/500 cycles	70/0.06	^[^ ^42^ ^]^
Ni/Fe LDH	–	2–3	1.7–2.8	844/1 C	501/1000 cycles	59.3/0.004	^[^ ^43^ ^]^
TCD‐TCS/S	67.6	1.8	1.5–3.0	1058/2 C	815/400 cycles	77.2/0.057	^[^ ^31^ ^]^
Ti_3_C_2_/S@PDA	78.3	1.5	1.7–3.0	1197/0.5 C	1096/200 cycles	91.6/0.042	^[^ ^44^ ^]^
ZDC@ZIF‐8	74.47%	–	1.6–2.8	1118/1 C	683/300 cycles	52/0.16	^[^ ^30^ ^]^
Chemisorption catalysis							
Co/N‐PCNF	62.2	2.0	1.7–2.8	878/1C	728/200 cycles	83/0.07	^[^ ^45^ ^]^
N‐PC@uCo	76	1.8	1.7–2.8	912/1 C	780/500 cycles	86/0.028	^[^ ^46^ ^]^
E‐Co*_x_* Sn*_y_*/NC	72	–	1.8–2.7	840/1 C	681/500 cycles	81.2/0.037	^[^ ^47^ ^]^
CoFe‐MCS	78.2	–	1.7–2.8	–/2C C	–/500 cycles	–/0.062	^[^ ^48^ ^]^
HCPT@COF	69.3	–	1.7–2.8	1149/0.5 C	875/800 cycles	76.2/0.03	^[^ ^49^ ^]^
a‐Ta_2_O_5‐_ *_x_*/MCN	66.2		1.8–2.6	–/1 C	–/1000 cycles	–/0.029	^[^ ^50^ ^]^
CNT@TiO_2‐_ *_x_*	70	≈2.2	1.7–2.8	–/1 C	598/500 cycles	–/–	^[^ ^51^ ^]^
NMRC/S@ MnO_2_	72	1.8	1.4‐2.8	1072/2 C	590/1000 cycles	55/0.045	^[^ ^52^ ^]^
CoS_2_/graphene	75	–	1.7–2.8	1003/2 C	321/2000 cycles	32/0.034	^[^ ^53^ ^]^
3DOM N‐Co_9_S_8‐_ *_x_*	69.4	–	1.8–2.6	1158/1	927.8/500 cycles	80/0.04	^[^ ^54^ ^]^
V‐MoS_2_‐CNF	–	2.0	1.7–2.6	1068/0.5	800/300 cycles	75/0.083	^[^ ^55^ ^]^
MoS_2‐_ *_x_*/rGO	78	–	1.8–2.6	1159.9/0.5	628.2/600 cycles	50.2/0.083	^[^ ^56^ ^]^
VS_4_@RGO	70	3.0	1.7–2.8	937/1 C	601/500 cycles	65/0.07	^[^ ^57^ ^]^
ZnSe/NHC	70.1	3.2	1.7–2.8	659/1 C	540.5/600 cycles	82/0.03	^[^ ^58^ ^]^
CC@CS@HPP	72	–	1.7–2.8	796.8/2 C	478/1000 cycles	60/0.04	^[^ ^59^ ^]^
Mo_2_C‐C NOs	72.15	–	1.7–2.8	1050/1 C	762/600 cycles	73/0.045	^[^ ^60^ ^]^
Fe_3‐_ *_x_* C@C‐500	74	–	1.7–2.8	–/1C	1000 cycles cycles	60.3/0.039	^[^ ^61^ ^]^
TSC/NbC	–	–	1.7–2.8	1287/0.1 C	1043/500 cycles	81.3/0.037	^[^ ^62^ ^]^
VN/G composite	–	3	1.7–2.8	1128/1 C	917/200 cycles	81/0.095	^[^ ^63^ ^]^
VN/N‐rGO	78.46	1.7	1.7–2.8	1101/0.5 C	959/500 cycles	87/0.026	^[^ ^64^ ^]^
C@TiN‐S	70	–	1.7–2.8	–/1 C	741/150 cycles	–/–	^[^ ^65^ ^]^
Fe_2_N@C	80		1.7–2.8	910/1 C	734/200 cycles	80.6/0.09	^[^ ^66^ ^]^
h‐Co_4_N@NC/S	75.5	1.5	1.0–3.0	786/5 C	658/400 cycles	83/0.04	^[^ ^67^ ^]^
				609/8 C	481/400 cycles	78/0.05	
Co_4_N	70	–	1.7–2.7	–/2 C	761/1000 cycles	–/–	^[^ ^68^ ^]^
				–/5 C	494/1000 cycles	–/–	
FeCFeOC	72	–	1.8–2.7	801/3 C	513/1000 cycles	64/0.036	^[^ ^69^ ^]^
TiO_2_‐TiN heterostructure	88	1.2	1.7–2.8	790/1 C	704/2000 cycles	89.1/0.005	^[^ ^70^ ^]^
		3.1		688/1 C	503/2000 cycles	73.1/0.013	
		4.3		523/1 C	331/2000 cycles	63.2/0.018	
TiO_2_–Ni_3_S_2_ heterostructure	80	–	1.7–2.8	980/0.5 C	638/500 cycles	65.1/0.06	^[^ ^71^ ^]^
CNF/PANi	67.1	2.0	1.7–2.8	629/0.2 C	711/300 cycles	–/–	^[^ ^72^ ^]^
SPANI	65	–	1.2–2.8	817/0.3 C	734/200 cycles	89.8/0.051	^[^ ^73^ ^]^

#### 3.1.1 Physisorption‐Confinement Effects

It is well known that the shuttle behavior of LiPSs is like a flow, shuttling from the high concentration side to the low one. Thus, confining the LiPSs in the cathode area by blocking or extending the diffusion paths of soluble LiPSs is the most direct and effective way to eliminate the shuttle phenomenon. To this end, host materials with novel nanostructured designs have been proposed for sulfur cathodes. And the most widely investigated hosts are carbonaceous hosts which exhibit special geometric space, large surface area, and good electrical conductivity. Serving as a polysulfides reservoir, these hosts can adsorb the LiPSs species physically through van der Waals force and confine them in the well‐designed porous space. Moreover, the porous carbon shell can act as a barrier to impede the dissolution of polysulfides.

##### Porous Carbon Matrices

**Figure 4:**
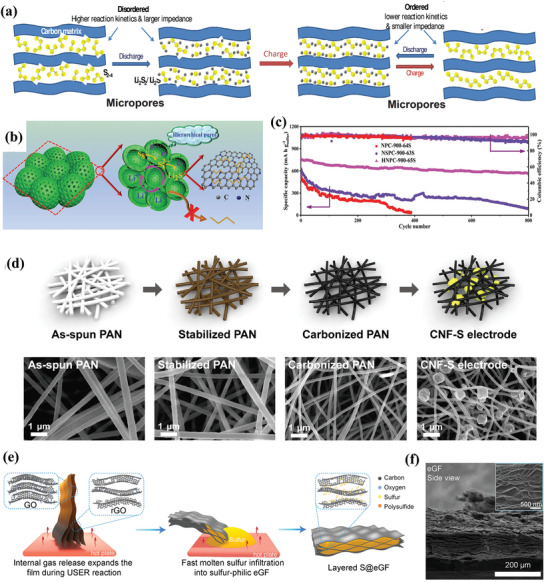
a) Schematic illustration of the structure change of the S_2–4_ molecules confined within micropores. Reproduced with permission.^[^ ^16^ ^]^ Copyright 2014, Wiley‐VCH. b) Schematic diagrams for HNPC architecture. c) Cycling stability performance of HNPC‐900‐65S at 2 C. Reproduced with permission.^[^ ^17^ ^]^ Copyright 2019, Wiley‐VCH. d) Fabrication of the CNF‐S electrode using electrospun PAN. Reproduced with permission.^[^ ^19^ ^]^ Copyright 2018, American Chemical Society. e) Schematic diagrams and f) SEM images for the expanded reduced graphene oxide film. Reproduced with permission.^[^ ^21^ ^]^ Copyright 2020, Elsevier.

The radius of the S_2–4_ chain is calculated to be ≈0.5 nm, while that of S_5–8_ chain is longer than 0.5 nm. Therefore, the micropores are regarded as ideal containers to store small sulfur species. Early in 2012, Wan and co‐workers reported the successful encapsulation of S_2–4_ sulfur within the conductive microporous carbon matrix with a pore size of ≈0.5 nm.^[^ ^15^ ^]^ In this system, S_2‐4_ cannot be transformed to long‐chain S_5–8_ due to the restriction of pore size, and the sulfur cathode with MPC host shows a single discharge plateau in charge and discharge curves and finally delivers an ultrahigh initial discharge capacity of 1670 mAh g^−1^, which is very close to the theoretical capacity. To fully understand the lithiation/delithiation mechanism of sulfur in microporous, Huang and co‐workers fabricated the S_2–4_ and S_8_/S_2–4_ composites with highly ordered microporous carbon as conductive matrices.^[^ ^16^ ^]^ Experimental results show that the short‐chain S_2–4_ occurs in a solid–solid process because the micropores of carbon are small enough to prevent the penetration of the solvent molecules and the formation of long‐chain sulfur species. And the smaller sulfur molecules show excellent cycle stability and better electrolyte compatibility (**Figure** **4**a). Finally, the solid‐state conversion process leads to a high coulombic efficiency (close to 100%) even in the absence of the LiNO_3_ additive. However, only a limited sulfur content (generally lower than 40%) could be achieved in the microporous carbon hosts, and they also have a lower voltage plateau, resulting in low energy density of the full battery, which are still puzzles for researchers. Compared with microporous carbon, the encapsulation of sulfur into the mesoporous carbon matrix can enhance the sulfur loading and the infiltration of electrolytes. For example, Li et al. reported the hollow core–shell carbon nanospheres with interfused architecture, which was derived from the pyrolysis of zeolitic imidazolate framework and polymer coating.^[^ ^17^ ^]^ Hierarchical pores ensure high content of sulfur and full penetration of electrolytes. Cross‐linked structures provide fast ions transport channels and the shuttling paths of LiPS are effectively blocked by the outer shell. As a result, such porous structured carbon hosts deliver a high capacity of 785 mAh g^−1^ at 2 C and 562 mAh g^−1^ is retained after 1000 cycles (Figure 4b,c).

##### Multidimensional Carbon Matrices

The subsequent iteration of carbon‐based hosts is developed along varied dimensions. Variants of 1D carbon‐based materials such as carbon nanotubes and carbon nanofibers have been sought to address the shuttle issues due to their high surface‐to‐volume ratio and large surface area. And 1D materials can serve as good conduction pathways for ions and electrons. In 2011, Cui and co‐workers have proved that hollow carbon nanofiber with high‐aspect ratio can serve as an ideal barrier for LiPSs diffusion.^[^ ^18^ ^]^ Similarly, the freestanding sulfur cathodes constructed by electrospun carbon nanofiber matrices were proposed by Lee et al.^[^ ^19^ ^]^ It was found that the solid sulfur and Li_2_S discharge product adhered well to the junction of the intertwined networks due to the cohesive force between the narrow gaps in the matrix (Figure 4d). Hence, the viscous polysulfides formed during cycling can be well trapped in this cross‐linked structure, which is very helpful to retard LiPSs dissolution. What is more, the 1D carbon matrix has a greater advantage on the volumetric changes compared to the electrical isolation of spherical carbon particles during Li–S batteries operation. As a result, a high areal capacity of 7.90 mAh g^−1^ was achieved under a high sulfur loading of 10.5 mg cm^−2^

Sulfur species can also be encapsulated within the interlayer of 2D carbon materials. Owing to the intrinsic high conductivity and laminar confining effect, 2D carbon‐based materials such as graphene can be served as barriers to suppress the shuttle effect.^[^ ^20^ ^]^ Chen et al. designed a horizontally arranged, high‐tortuosity porous reduced graphene oxide (rGO) for efficient sulfur hosts.^[^ ^21^ ^]^ These horizontally arranged rGO sheets constructed a mezzanine space to confine the dissolved LiPSs. Besides, experimental results show that the inhibitory effect of LiPSs diffusion and dissolution is positively correlated with the tortuosity degree of graphene oxide. The higher electrode tortuosity could contribute to the geometrically extended outward mass transport pathways to suppress outward LiPSs diffusion from the cathode (Figure 4e,f). With these integrated merits, an ultrahigh cathode areal capacity of 21 mAh cm^‐2^ with 98.1% of capacity retention was obtained after 160 cycles. Following this line of thought, the core concept of confining the sulfur within a conductive matrix can be further applied to design 3D framework hosts. For instance, a dense graphene/sulfur composite cathode was obtained by shrinking the sulfur‐loading graphene foam through surface tension by Zhang and co‐workers.^[^ ^22^ ^]^ In this structure, soluble LiPSs are restrained within the closed pores, thus the shuttle phenomenon is suppressed effectively, eventually achieving high volumetric energy density and long‐term cycling performance. In addition, the self‐assembled sulfur cathode with a 3D structure can be directly used as freestanding electrodes for Li–S batteries, such as 3D graphene and sulfur nanocrystals, 3D graphene foam, and so on.^[^ ^23^ ^]^

#### 3.1.2 Adsorption Effects Extension

Although the soluble LiPSs can be adsorbed and encapsulated within the carbon host through the van der Waals force and confinement effects, the shuttle behavior of LiPSs could be suppressed to some extent. In other words, the movement and diffusion of LiPSs cannot be eliminated fundamentally based on weak physical interaction. Further controlling the interaction way is crucial to effectively enhance the anchor ability toward LiPSs. In light of this, the utilization of chemical adsorption by electron transfer, exchange, or sharing to form adsorption chemical bonds with atoms (or molecules) on the solid surface, has drawn great research interest. Chemical adsorption shows stronger adsorbability than physical effects, thus, the polysulfides will be more difficult to escape from conductive matrices. According to the interaction mode, the chemical adsorption between sulfur hosts and polysulfides is divided into two categories: polar‐polar interactions and Lewis acid–base interactions.

##### Polar–Polar Interactions

**Figure 5:**
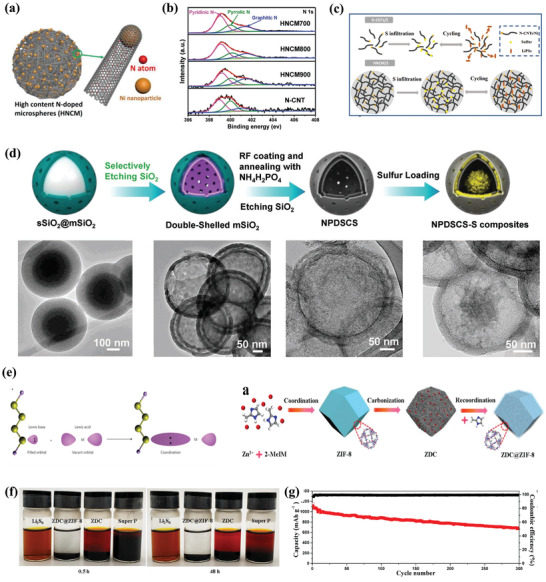
a) Schematic illustration and b) XPS spectra of N 1s of HNCM. c) Schematic illustration of the operating principle for HNCM and N‐CNTs hosts. Reproduced with permission.^[^ ^24^ ^]^ Copyright 2019, Wiley‐VCH. d) Schematic illustration of the fabrication process of NPDSCS‐S and the corresponding TEM images. Reproduced with permission.^[^ ^27^ ^]^ Copyright 2018, Wiley‐VCH. e) Schematic of the fabrication of ZDC@ZIF‐8. f) Visualization adsorption test of ZDC@ZIF‐8 in Li_2_S_6_ solution. g) Cycling stability of the S/ZDC@ZIF‐8 cathode at 1 C for 300 cycles. Reproduced with permission.^[^ ^30^ ^]^ Copyright 2018, Elsevier.

Considering the polarity of polysulfides, materials with strong polarity have the potential as good sulfur hosts. The design of polar hosts, e.g., modification of the porous carbon matrix via heteroatoms doping or the introduction of polar substance can entrap the soluble LiPSs effectively through polar–polar interactions. Of which, heteroatom doping is regarded as one of the most efficient ways to enhance the surface polarity of nonpolar carbon substrates. Wang et al. proposed the high‐content N‐doped carbon nanotube microspheres (HNCMs) for LiPSs immobilizing.^[^ ^24^ ^]^ Pyridinic and pyrrolic nitrogen possess an extra pair of electrons and they can absorb the polar LiPSs via a coordination bond‐like mode between Li atoms. When evaluated as a sulfur host, the high content of 12.43 at% nitrogen doping offers a strong chemical affinity toward LiPSs and entraps them within the microspheres tightly (**Figure** **5**a–c). As a result, the optimized sulfur cathode delivers an initial specific capacity of 902 mAh g^‐1^ and retains a high specific capacity of 804 mAh g^‐1^ after 1000 cycles at 0.5 C. Apart from that, heteroatom‐doping is not limited to nitrogen but many types of doping elements such as O, S, P, Se, and so on.^[^ ^25^ ^]^ Zhou et al. have designed the nitrogen/oxygen dual‐doped hollow carbon microspheres (HCMs) and 5.36 at% of nitrogen and 6.99 at% of oxygen were doped in the carbon skeleton.^[^ ^26^ ^]^ The dual‐doping strategy could afford more powerful anchor sites for LiPSs. Similarly, nitrogen and phosphorus co‐doped carbon spheres with double‐shelled structures have also been constructed by Shen and co‐workers.^[^ ^27^ ^]^ Porous double‐shelled and hollow structure provides sufficient space to host sulfur and suppress the dissolution of polysulfides intermediates. And the doping of nitrogen and phosphorus can enhance the affinity and trapping ability toward LiPSs (Figure 5d).

Compared with carbon materials, the richness in oxygen, nitrogen, and sulfur atoms in metal‐based materials naturally offers a strong affinity for LiPSs. Among them, metal oxides have been widely reported to confine polysulfides by polar–polar interactions. Metal–sulfur or oxygen–lithium bonds could be formed based on the strong polarity. Cui's group initially proposed the hydrogen‐reduced TiO_2_ to enhance the binding with the LiPSs, and a high capacity retention of 81% was achieved after 200 cycles at 0.2 C.^[^ ^28^ ^]^ Subsequently, the Magnéli phase titanium oxide Ti_4_O_7_ was further discovered, in which Ti_4_O_7_ containing polar O–Ti–O units has a strong affinity for polysulfides. The strong metal oxide‐polysulfide chemical interactions were confirmed through the visual adsorption investigations with X‐ray photoelectron spectroscopic (XPS) and X‐ray absorption near‐edge structure (XANES) studies. Inspired by earlier studies, other oxides including MoO_2_, Co_3_O_4_, and Fe_3_O_4_ are also developed to anchor polysulfides.^[^ ^29^ ^]^ Furthermore, many other materials hosts such as sulfides, nitrides, and carbides, with high polarity have been widely investigated. Notably, some of them exhibit not only strong adsorption performance but also excellent catalytic activity, which will be discussed in detail in the following chemisorption‐catalysis section.

##### Lewis Acid–Base Interactions

The polysulfide anions (S*_x_* ^2–^, 4 ≤ *x* ≤ 8) own occupied orbitals with lone electron pairs, which can be considered as a Lewis base. And they can provide redundant electronic pairs for Lewis acid to form coordinate bonds. Therefore, the soluble LiPSs can be fixed by host materials with Lewis acid peculiarity. Metal ions in metal‐organic frameworks (MOFs) and MXenes are considered as the most representative Lewis acid sites that can anchor the LiPSs efficiently through Lewis acid–base interactions. Mai and co‐workers reported a zeolitic imidazolate framework‐8 (ZIF‐8) coated polyhedral carbon matrix (ZDC@ZIF‐8) to alleviate the shuttle effect.^[^ ^30^ ^]^ Exempting to the strong acid wash step, the new outer ZIF‐8 layer was fabricated by the ZnO nanoparticles in the pyrolytic polyhedral that derived from the original ZIF‐8 precursor (Figure 5e). According to the adsorption test, Li_2_S_6_ solution with ZDC@ZIF‐8 becomes colorless after aging half an hour while the other two samples with ZDC and Super P are still yellow after aging 48 h, confirming the strong chemical interactions of metal ions and LiPSs (Figure 5f). And the resulting cathode displays a high initial capacity of 1118 mAh g^−1^ and retains 683 mAh g^−1^ after 300 cycles (Figure 5g). Similarly, as nascent 2D metal materials, MXenes have recently captured considerable attention in Li–S batteries owing to their excellent electronic conductivity and rich surface functional groups. Xiao et al. reported a unique Ti_3_C_2_T*_x_* (TC) nanodots‐TC nanosheets (TCD‐TCS) sulfur host to accomplish the spatial immobilization and chemisorption to the LiPSs.^[^ ^31^ ^]^ Soluble LiPS can be trapped effectively by the high density of surface polar sites. And the intimate connection between isogenous nanodots and nanosheets could decrease their interfacial resistance greatly. Consequently, the conductive additive‐free sulfur cathode delivers high areal capacities of 13.7 mAh cm^−2^ at ultrahigh area sulfur loadings of 13.8 mg cm^−2^.

#### 3.1.3 Chemisorption‐Catalysis Effects

Gaining deep insight into the multistep and sluggish conversion mechanism of the intermediate LiPSs, accelerating the conversion process of LiPSs is thus a crucial way to avoid the accumulation of soluble NaPSs and alleviate the severe shuttle effect. Inspired by traditional catalysis, electrocatalysis effects have been introduced for Li–S batteries in recent years. And numerous studies have been carried out to accelerate the conversion speed and shorten the residence time of long‐chain LiPSs in the electrolyte with chemical adsorption and electrocatalysis effects synergistically.^[^ ^74^ ^]^ To be specific, when the electrocatalyst is added to the sulfur electrode, the soluble LiPSs will be adsorbed by electrocatalysts firstly. Adsorption is a prelude to electrocatalysis, the dissociative LiPSs would not be effectively fixed to the electrocatalyst surface if the adsorption strength is weak, resulting in reduced conversion efficiency.^[^ ^75^ ^]^ Afterward, the conversion speed enhancement is governed by the charge‐transfer kinetics, which is closely related to the intrinsic conductive properties and catalytic activity of substrate materials.^[^ ^75^, ^76^ ^]^ On this topic, it is worth noting that most of the so‐called “catalysts” in Li–S batteries are quite different from those conventional chemical catalysts, which might change the surface state or composition during the catalytic process.^[^ ^77^ ^]^ To distinguish, the term “mediator” is often used to categorize those host materials that can promote the polysulfides conversion in Li–S chemistry.^[^ ^78^ ^]^ In the following section, the recent research progress of such novel sulfur hosts with chemisorption‐catalysis effects are introduced retrospectively and discussed in detail.

##### Metals and Single‐Atoms Catalysts

**Figure 6:**
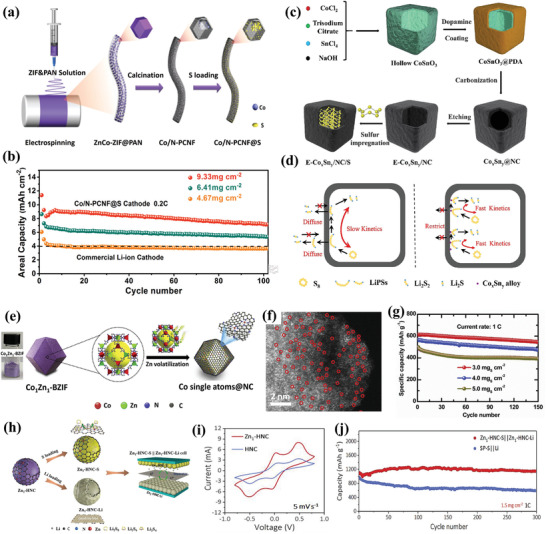
a) Schematic illustration of the fabrication for the Co/N‐PCNF@S composite. b) Areal capacities of the N‐PCNF@S cathode with different sulfur loading at 0.2 C. Reproduced with permission.^[^ ^45^ ^]^ Copyright 2020, Royal Society of Chemistry. c) Schematic illustration of the preparation for E‐Co*_x_* Sn*_y_*/NC/S composite. d) The schematic illustration of the lithiation/delithiation process for the E‐Co*_x_* Sn*_y_*/NC/S cathode without Co*_x_* Sn*_y_* (left) and with Co*_x_* Sn*_y_* alloy (right). Reproduced with permission.^[^ ^47^ ^]^ Copyright 2019, Elsevier. e) Schematic illustration of synthesis processes and f) corresponding SEM image of Co‐SAs@NC. g) Cycling performance of S/Co‐SAs@NC at different sulfur loading. Reproduced with permission.^[^ ^83^ ^]^ Copyright 2020, Elsevier. h) Schematic illustration for the synthesis of dual‐functional Zn_1_‐HNC nanoreactors. i) CV curves of Li_2_S_6_ symmetric cells. j) Cycling stability at 1.0 C of Zn_1_‐HNC‐S||Zn_1_‐HNC‐Li and SP‐S||Li batteries. Reproduced with permission.^[^ ^84^ ^]^ Copyright 2020, Wiley‐VCH.

In Li–S batteries field, Arava and co‐workers initially investigated the catalytic activity of Pt to promote the LiPSs conversion and found that the prepared Pt/graphene hybrid delivered a stronger catalytic activity than Ni/graphene counterpart.^[^ ^79^ ^]^ Beyond noble metal materials, Pan and co‐workers designed a nitrogen‐doped porous carbon with cobalt clusters (N‐PC@uCo) for sulfur redox kinetics promotion.^[^ ^46^ ^]^ The highly dispersed Co clusters can trap the dissociative LiPSs efficiently. And the Li^+^ ions transfer rate is significantly promoted due to the addition of Co clusters. Recently, our group has designed a 3D interconnected Co‐decorated and N‐doped porous carbon nanofiber (Co/N‐PCNF) network, serving as a freestanding and high loading sulfur cathode for Li–S batteries (**Figure** **6**a).^[^ ^45^ ^]^ Polar Co nanoparticles possess strong chemisorption and excellent catalytic ability toward LiPSs, which can anchor the LiPSs strongly and further accelerate their conversion. Moreover, 3D cross‐linked conductive networks can facilitate the infiltration of electrolyte and electronic transport. Consequently, even under the high sulfur loading of 9.33 mg cm^−2^, the as‐prepared cathode remained a high areal capacity of 7.16 mAh cm^−2^ after 100 cycles at 0.2 C (Figure 6b). Aside from the single metal component, bimetallic alloys can promote LiPSs conversion synergistically through the synergistic effect of metal‐metal interactions. Hollow N‐doped carbon nano‐boxes modified by Co*_x_* Sn*_y_* (E‐Co*_x_* Sn*_y_*/NC) were proposed by Qiao et al.^[^ ^47^ ^]^ In such a hollow structure, dissolved LiPSs can be confined in the inner void space (Figure 6c). The visualized experiments show that The E‐Co*_x_* Sn*_y_*/NC‐3 electrode exhibits a stronger affinity to LiPSs than its control counterparts because the color of polysulfides solution became transparent more quickly. And, the higher response current in the symmetrical cell further certified the strong electrocatalytic ability to accelerate the redox kinetics of LiPSs (Figure 6d). As a result, the assembled cathode maintained capacity retention of 81.2% after 500 cycles at 1.0 C and delivered a stable areal capacity of 4.08 mAh cm^−2^ over 100 cycles with high sulfur loading of 4.3 mg cm^−2^. In addition, the other bimetallic catalysts such as CoFe^[^ ^80^ ^]^ and Fe–Ni^[^ ^81^ ^]^ are all favorable for polysulfides shuttle inhibition.

In the recent past, single‐atom catalysts (SACs) with well‐dispersed metal atoms, 100% of atom‐utilization efficiency, and unique coordinate environment have emerged as a new frontier in Li–S catalysis science.^[^ ^82^ ^]^ Zhou et al. have screened a series of SAC materials for catalyzing decomposition of Li_2_S by theoretical simulation.^[^ ^82^ ^]^ The authors found that the vanadium single atoms on N‐doped graphene (SAV@NG) possess the biggest binding energy of 3.38 eV toward Li_2_S_6_ and the smallest decomposition barrier (1.10 eV) for Li_2_S, indicating that the SAV@NG material exhibits the best potential for promoting the LiPSs conversion. Liu and co‐workers prepared the N‐doped carbon dodecahedra decorated with cobalt SAs (Co‐SAs@NC) by calcining the Co–Zn bimetallic metal‐organic framework precursor (Figure 6e).^[^ ^83^ ^]^ In this way, the Zn component in the precursor partially occupies the coordination sites of Co and further provides open sites for N implanting after evaporation (Figure 6f). Benefiting from the rich and highly dispersed Co‐SAs catalytic sites, the assembled cell with S@Co‐SAs@NC cathode could operate for 150 cycles without significant capacity decay under 5 mg cm^‐2^ of sulfur loading (Figure 6g). In another report, Wu and co‐workers designed single atom zinc‐decorated hollow carbon nanoreactor derived from ZIF‐8, which was employed as sulfur and lithium host simultaneously in Li–S full cells (Figure 6h).^[^ ^84^ ^]^ In this system, the nanoreactor with atomic zinc decoration cannot only accelerate polysulfides conversion but also guide the uniform deposition of Li metal owing to their high surface area and abundant active sites (Figure 6i). As a result, the assembled full cell based on this dual‐functional host delivers a stable discharge capacity of 1149 mAh g^−1^ after 300 cycles at 1 C (Figure 6j). In addition, Fe SACs also show an excellent catalytic effect, the Fe SAs supported on N‐doped holey graphene could help to boost the LiPSs conversion and enhance the cycling stability.^[^ ^85^ ^]^

##### Metal Oxides

**Figure 7:**
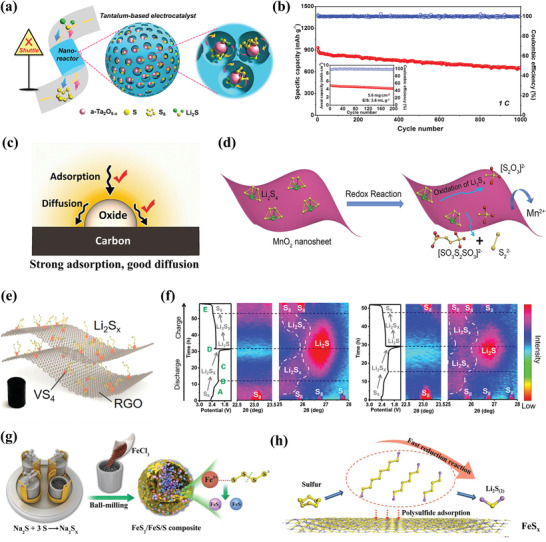
a) Scheme illustration of LiPSs catalytic conversion in a‐Ta_2_O_5‐_ *_x_*/MCN nanoreactor. b) Cycling performance of a‐Ta_2_O_5‐_ *_x_*/MCN/S cathode. Reproduced with permission.^[^ ^50^ ^]^ Copyright 2020, Elsevier. c) Schematic illustrations of the LiPSs adsorption and diffusion on nonconductive metal oxides surface. Reproduced with permission.^[^ ^87^ ^]^ Copyright 2016, Nature Publishing Group. d) The interaction mechanism between polysulfides and MnO_2_. Reproduced with permission.^[^ ^52^ ^]^ Copyright 2020, Elsevier. e) Schematic illustration of the VS_4_@RGO framework. f) In situ XRD patterns in contour plots of the Li–S cells with (left) and without (right) VS_4_. Reproduced with permission.^[^ ^57^ ^]^ Copyright 2020, American Chemical Society. g) The synthesis process of FeS_2_/FeS/S composites. h) The adsorption mechanisms of polysulfides onto the surface of FeS*_x_*. Reproduced with permission.^[^ ^93^ ^]^ Copyright 2019, Wiley‐VCH.

Metal oxides possess a high chemical affinity toward polar LiPSs due to their strong polar surface and rich hydrophilic oxygen groups. and the excellent electrocatalytic properties endow them with great prospective for shuttle effect inhibition.^[^ ^86^ ^]^ Recently, Zhang et al. dexterously designed the amorphous tantalum oxide with oxygen vacancies embedded in the microporous carbon matrix (a‐Ta_2_O_5‐_ *_x_*/MCN), serving as an efficient electrocatalyst for LiPSs.^[^ ^50^ ^]^ As shown in **Figure** **7**a, the “‘ship in a bottle”’ structure can accommodate a large amount of active sulfur. And the oxygen deficiencies in Ta_2_O_5_ nanoclusters could significantly improve the inherent electronic conductivity and function as catalytic centers to anchor the soluble LiPSs. The highest current response and lowest onset potential in Linear sweep voltammetry (LSV) curves indicate the lowest energy barrier for oxidation conversion and superior catalytic activity of a‐Ta_2_O_5‐_ *_x_*/MCN hosts. Consequently, the resulting sulfur cathode achieved a long‐term cyclability over 1000 cycles with an ultralow capacity fading rate of 0.029% per cycle and a high areal capacity of 5 mAh cm^−2^ under sulfur loading of 5.6 mg cm^−2^ (Figure 7b). However, it is worth noting that most metal oxides are not suitable to be used as a sulfur host directly due to the nature of insulation. So the metal oxides are always integrated with conductive substrates to the improve the conductivity. In 2016, Cui and co‐workers have elucidated that the adsorbed LiPSs need to migrate to the boundary interface between metal oxides and conductive substrates to accomplish the charge transfer.^[^ ^87^ ^]^ Thus, the anchor ability and diffusion barrier need to be considered simultaneously for designing sulfur hosts with metal oxides (Figure 7c).

So far, various kinds of metal oxides have been employed as sulfur hosts for Li–S batteries, such as Fe_3_O_4_, ^[^ ^88^ ^]^ MnO_2_, ^[^ ^89^ ^]^ Al_2_O_3_,^[^ ^90^ ^]^ and SiO_2_. ^[^ ^91^ ^]^ Particularly, some metal oxides with a redox potential within the targeted range, such as MnO_2_, which can first react with LiPSs to form the thiosulfate (S_2_O_3_^2−^), then the S_2_O_3_^2−^ continue to react with LiPSs to generate the polythionate complex [O_3_S_2_‐(S)*_x_*_−2_‐S_2_O_3_]^2–^. Consequently, the shuttle phenomenon of LiPSs can be suppressed to some extent owing to the poor solubility of polythionate.^[^ ^92^ ^]^ In vein of this, Su et al. designed a double chemisorption cathode via the in situ growth of MnO_2_ nanosheets on the hollow nitrogen‐doped micropore rich carbon (NMRC).^[^ ^52^ ^]^ According to the XPS analysis, the thiosulfate groups were generated on the surface of MnO_2_ and the thiosulfates could adsorb the newly formed polysulfides to form polythionate complexes. These polythionates can serve as new mediators to accelerate redox kinetics and improve cycling performance (Figure 7d).

##### Metal Sulfides

Metal sulfides possess strong sulfiphilic properties and relatively low lithiation potential, which endow them with excellent electrocatalysis for the redox reactions in Li–S batteries. Moreover, metal sulfides generally exhibit higher conductivity than oxides and some metal sulfides even possess metallic or half‐metallic phases. As a representative study, Zhang and co‐workers initially demonstrated the electrocatalysis of CoS_2_ in Li–S chemistry with a high capacity of 1003 mAh g^−1^ was achieved in the first cycle and an average capacity decay rate of 0.034% per cycle after 2000 cycles at 2 C.^[^ ^53^ ^]^ Recently, Manthiram's group designed a novel sulfur host with vanadium tetrasulfide embedded in a reduced graphene oxide framework (VS_4_@RGO).^[^ ^57^ ^]^ The porous framework could serve as reservoirs to accommodate sufficient sulfur and the uniformly dispersed sulfiphilic VS_4_ nanoparticles act as polysulfide mediators to offer sufficient polysulfides anchoring and catalytic sites (Figure 7e). Notably, the in situ XRD pattern of the cell with VS_4_ additive delivers weaker polysulfides signals at the full discharged state, whereas the sulfur cathode without VS_4_ additive shows stronger signals, indicating the less content of polysulfides in the electrolyte (Figure 7f). Finally, the assembled sulfur cathode with VS_4_@RGO approaches an initial discharge capacity of 937 mAh g^−1^ at 1 C and maintains a high reversible discharge capacity of 601 mAh g^−1^ after 500 cycles, which were much higher than the sulfur cathode without VS_4_@RGO additive. Xi et al. prepared the FeS_2_/FeS/S composite by a simple ball‐milling method and yielded a high tap density, high volumetric capacity, and a short electron pathway (Figure 7g).^[^ ^93^ ^]^ In this system, two configurations of Li_2_S*_x_* chain adsorption are detected according to the DFT calculation, and in‐plane adsorption is more energetically favorable than perpendicular adsorption. Besides, the calculated binding energies for adsorption onto FeS_2_ are shown to be consistently larger than those onto FeS. High‐order polysulfides tended to form into two shorter chain segments when adsorbed onto FeS_2_ by the catalytic cleavage ability. From another investigation, Boyjoo et al. prepared the Fe_1‐_ *_x_* S nanoparticles embedded in hierarchically porous N‐doped carbon spheres for enhancing the polysulfides redox kinetics.^[^ ^94^ ^]^ The nanoreactors possess low mass density because of the high porosity, and highly dispersed Fe_1‐_ *_x_* S nanoparticles can effectively trap the soluble LiPSs and catalyze their conversion. Consequently, the resulting sulfur cathode delivers a high initial capacity of 1070 mAh g^−1^and excellent cycling stability.

##### Metal Carbide and Metal Nitrides

**Figure 8:**
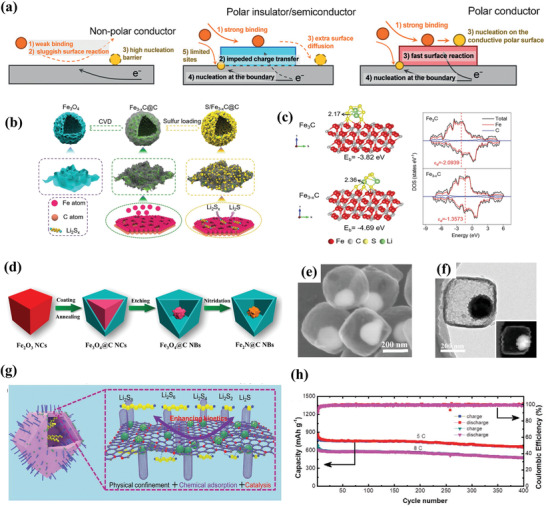
a) Schematic illustration of the working mechanism for different conductors with different types of binding and charge transfer. Reproduced with permission.^[^ ^96^ ^]^ Copyright 2016, Wiley‐VCH. b) Schematic illustration of the synthesis for the S/Fe_3‐_ *_x_* C@C. c) Geometrically stable configurations and DOS state of Li_2_S_6_ adsorption on Fe_3_C (031) and Fe_3‐_ *_x_* C (031) surfaces. Reproduced with permission.^[^ ^61^ ^]^ Copyright 2020, Wiley‐VCH. d) Schematic illustration of the fabrication process of yolk‐shelled Fe_2_N@C NBs. e) SEM image of the produced Fe_2_N@C NBs. f) TEM image of S/Fe_2_N@C NBs. Reproduced with permission.^[^ ^66^ ^]^ Copyright 2019, Wiley‐VCH. g) Synthesis process of the h‐Co_4_N@NC/S electrode. h) Cycling performance of the h‐Co4N@NC/S composite. Reproduced with permission.^[^ ^67^ ^]^ Copyright 2020, Wiley‐VCH.

Polar metal carbides and metal nitrides have captured intensive attention in the field of catalysis and energy storage field owing to their inherently excellent conductivity, favorable thermal stability as well as similar catalytic properties akin to noble metals.^[^ ^95^ ^]^ For Li–S batteries, Zhang and co‐workers reported the TiC as a conductive polar redox mediator to increase the intrinsic catalytic activity with adequate binding affinity and efficient charge transfer kinetics (**Figure** **8**a).^[^ ^96^ ^]^ In this system, nanosized TiC/mesoporous graphene exhibited a great improvement in both the reversible interfacial redox of LiPSs and the precipitation of Li_2_S owing to the strong adsorbability and excellent conductivity. From another investigation, the unique hierarchical Fe_3‐_ *_x_* C@C hollow microspheres with enriched Fe‐vacancies were designed by Chen and co‐workers (Figure 8b).^[^ ^61^ ^]^ The Fe_3‐_ *_x_* C exhibits a much higher Li_2_S_6_ adsorption energy than that on the Fe_3_C demonstrating the resultant high‐efficiency adsorption behavior of Fe_3‐_ *_x_* C (Figure 8c). Moreover, hierarchical porous architecture not only can accommodate sufficient sulfur but also establish favorable electron/ion transfer highways. Hence, the modified sulfur cathode displayed a high specific capacity and excellent cycle stability.

Similar to metal carbides, metal nitrides are prospective mediator candidates for Li–S batteries. The lone electron pairs in metal nitrides can function as Lewis base matrixes to chemically interact with LiPSs and anchor them.^[^ ^68^ ^]^ Among them, VN exhibits a significant role in Li–S batteries due to the intriguing high electronic conductivity of about 10^6^ S m^−1^ at room temperature.^[^ ^97^ ^]^ Zhong et al. designed a porous carbon fiber/VN (PCF/VN) integrated scaffold as a sulfur cathode with high conductivity and a smooth catalysis‐conduction interface.^[^ ^98^ ^]^ In such a structure, soluble LiPSs can be absorbed strongly on the surface owing to the synergistic dual bonding (Ti‒S and Li‒N). Sun et al. have successfully synthesized the iron‐based nitride for Li–S batteries (Figure 8d).^[^ ^66^ ^]^ A unique yolk‐shelled carbon nanobox structure was obtained by etching and nitridation treatment. More negative values of corresponding binding energies toward sulfur species demonstrate the stronger adsorption with LiPSs than that of Fe_3_O_4_. And the robust carbon framework can alleviate the volume variation of sulfur substances and impede the shuttle behavior of LiPSs physically (Figure 8e,f). As expected, a low fading rate of 0.036% per cycle with 881mAh g^−1^ was observed at 1 C after 600 cycles. Analogously, Li and co‐workers proposed the N‐doped double‐shelled hollow carbon cage with Co_4_N decoration to obtain physiochemical confinement effects (Figure 8g).^[^ ^67^ ^]^ The soluble LiPSs could be spatially confined in this hollow structure to mitigate the movement phenomenon. Combining the efficient pathways for charge transfer and the strong chemical binding with the polysulfides, the as‐prepared electrode maintained a high reversible capacity of 658 and 481 mAh g^−1^ at 5 C and 8 C after 400cycle, respectively (Figure 7h).

##### Heterostructures and Hybrids

**Figure 9:**
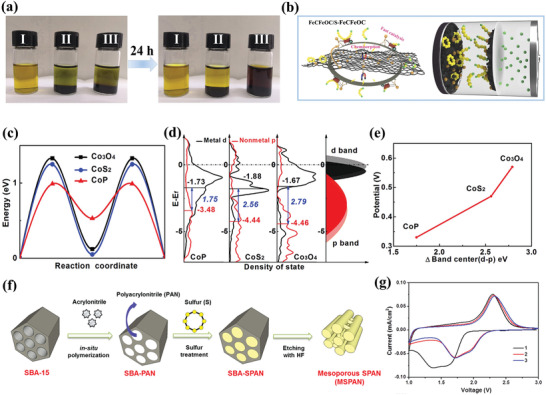
a) The optical photographs of adsorption measurement. b) Schematic illustrations of discharge–charge processes for FeCFeOC composite. Reproduced with permission.^[^ ^69^ ^]^ Copyright 2021, Wiley‐VCH. c) Diffusion energy barriers of Li_2_S on different substrates. d) Density of states analysis of the p bands of anions and the d band of Co in different Cobalt‐based compounds. e) Scaling relation between the D band (d‐p) center and Li–S redox potentials. Reproduced with permission.^[^ ^102^ ^]^ Copyright 2018, Elsevier. f) Schematic illustration of the sequential fabrication steps for MSPAN composite. g) CV profile of MSPAN cell at a scan rate of 0.05 mV s^−1^. Reproduced with permission.^[^ ^106^ ^]^ Copyright 2017, American Chemical Society.

To integrate the advantages of multiple materials, hybrid designs, such as heterostructure hosts which combine strong adsorbability and good catalysis ability have been customized for Li–S batteries.^[^ ^99^ ^]^ In such systems, a smooth trapping‐diffusion‐conversion path for polysulfides can be obtained from the synergistic effect of multiple materials. In this way, a TiO_2_–Ni_3_S_2_ heterostructure was designed by Yang et al. based on the strong adsorbability of TiO_2_ and the fast electron transfer ability of Ni_3_S_2_.^[^ ^71^ ^]^ During the reduction process, TiO_2_ provided a strong capturing effect on soluble polysulfides, and Ni_3_S_2_ catalyzed the reduction process and regulated the deposition of solid Li_2_S. Recently, our group has successfully designed a novel multiphase Fe‐based compounds (including Fe_3_C, Fe_3_O_4_, and Fe_2_O_3_, denoted as FeCFeOC) embedded into a 3D carbon network. This multiple‐phase compound was employed as a sulfur cathode host and separator modified layer.^[^ ^69^ ^]^ In this system, 3D interconnected networks secure a large amount of active sulfur and mitigate the volume variation simultaneously. The transfer of electronic and Li^+^ ions was further significantly promoted by the conductive Fe_3_C. Interestingly, the color of Li_2_S_6_ solution containing FeCFeOC composite changed from yellow to dark while the other one containing the bare carbon framework was kept almost unchanged in the adsorption test (**Figure** **9**a). After systematic exploration, we found that this phenomenon was ascribed to the spontaneous chemical reaction between Fe*_x_* O*_y_* and LiPSs, leading to the generation of the magnetic FeS*_x_* species with larger sizes, which can impede the diffusion of the dissociative LiPSs (Figure 9b). As excepted, the assembled Li–S cells showed excellent long‐term stability with 748 mAh g^−1^ over 500 cycles at 1.0 C. Additionally, the other hybrids such as Co_9_S_8_/CoO,^[^ ^100^ ^]^ MoO_3_/MoO_2_,^[^ ^29a^ ^]^ Co_9_S_8_@MoS_2_,^[^ ^101^ ^]^ and WS_2_–WO_3_^99^ have also been investigated to enhance the redox kinetics. Overall speaking, it is anticipated that these ameliorative strategies by combining the strong adsorption and high conductivity catalyst hosts would be ideal models to enhance the polysulfides redox kinetics thus inhibiting the shuttle effect efficiently.

##### Other Emerging Metal‐Based Mediators

With the continuous upgrading of nanosynthesis technology and the improvement of characterization technology, a series of emerging redox mediators have been proposed to promote the reaction kinetics of Li–S batteries such as metal phosphides,^[^ ^102^ ^]^ metal selenides,^[^ ^59^ ^]^ metal borides,^[^ ^103^ ^]^ defect engineerings,^[^ ^104^ ^]^ and so on. Among them, Qian and co‐workers found that the p band of P anions in CoP exhibit a more distinct upshift toward the Fermi level than the other counterparts according to the DFT calculation, which reduces the energy gap between the cobalt 3d and anion 2p band centers (Figure 9c–e).^[^ ^102^ ^]^ Hence, CoP hosts deliver a high reversible capacity and stable cyclic ability on account of the moderate adsorption ability and superior diffusion dynamics. In addition, metal selenides possess similar crystallographic structures and polar characteristics to metal sulfides, but metal selenides usually exhibit higher conductivity than oxides and sulfides counterparts.^[^ ^105^ ^]^ By utilizing a facile solidoid selenylation process, Ye et al. have successfully synthesized the CoSe electrocatalyst with hierarchical porous nano‐polyhedron architecture.^[^ ^59^ ^]^ Hollow nanoreactor architecture impedes the diffusion path of soluble LiPSs, and the polar CoSe can catalytically accelerate the diffusion/conversion of polysulfides and precipitation/decomposition of Li_2_S.

To sum up, despite the outstanding electrochemical performances that have been achieved by the utilization of a variety of metal‐based compound sulfur hosts, there are still many key issues that need to be addressed before their practical applications. First of all, Although the electrocatalysis for LiPSs has been deeply rooted in the concept of Li–S chemistry, the mechanism of the catalytic reaction is still not clear enough, and the evolution of the catalyst is ambiguous, remaining a lot of doubts and space for further exploring. Besides, the extensive use of conductive carbon for enhancing the conductivity of non‐ or poor‐conductive metal compounds, such as metal oxides, will sacrifice the overall volumetric energy density. Furthermore, the synthesis of some metal‐based host materials usually involves too complicated or cumbersome preparation processes. For example, the synthetic process of metal nitrides usually involves the usage of NH_3_ and high‐temperature treatment, hindering their broad applications. Therefore, facile, controllable, and viable synthesis methods are urgent need to promote the broad application of those efficient metal‐based catalysts.

#### 3.1.4 Other Types of Sulfur Cathodes

Polymers have long been considered as promising sulfur hosts owing to their diverse surface functional groups and tailorable electrical properties. Among them, some polymers exhibit excellent electronic conductivity which can serve as conductive coating materials or as sulfur hosts directly. For instance, Cui and his co‐workers initially modified the CMK‐3 mesoporous carbon/sulfur with conductive polymer poly (3,4‐ethylene dioxythiophene)‐poly (styrene sulfonate) (PEDOT:PSS) to minimize the dissolution of LiPSs.^[^ ^107^ ^]^ And then, they further employed different polymer‐coated hollow sulfur nanospheres to systematically investigate the confining effect of conductive polymers in inhibiting the shuttle effect.^[^ ^108^ ^]^ It was found that the polymer shells were able to restrict the LiPSs physically and the surface functional groups exhibited strong chemical interaction to LiPSs, thus improving the cycling stability and rate capability. In addition, adding sulfur into polymer materials to form molecular‐level composites through covalent bonding can prevent the formation of long‐chain LiPSs and suppress the shuttle effect completely. In 2002, Wang and co‐workers initially proposed sulfurized polyacrylonitrile (SPAN) by heating the mixture of acrylonitrile and active sulfur.^[^ ^109^ ^]^ From another recent report, mesoporous sulfurized polyacrylonitrile (MSPAN) cathode was prepared by using the ordered mesoporous SBA‐15 as a template.^[^ ^106^ ^]^ As shown in Figure 9f, PAN was in situ polymerized to generate SBA‐PAN, and sulfur was incorporated into the PAN chains by the following sulfurization process. The MSPAN possessed a large specific surface area and highly ordered mesoporous structure, which could improve the electrolytes infiltration and facilitate electronic and ionic transport. Noticeably, the MSPAN cells exhibited a pair of cathodic peaks at 1.7 and 2.0 V in CV curves, indicating the absence of long‐chain LiPSs during cycling (Figure 9g). This unique reaction mechanism enabled such cathode material to obtain excellent cyclic stability and excellent rate capability. Thus, the MSPAN electrode exhibited exceptionally excellent electrochemical performance with a reversible capacity of around 610 mAh g^‐1^ after 900 cycles at 2 C. However, these SPAN‐based cathodes are always limited by the insufficient sulfur content (usually less than 50 wt%), which is hard to meet the high energy characteristics of practical Li–S batteries. Therefore, it is of great significance to increase the sulfur content in these polymers‐based cathodes without sacrificing the excellent cyclic performance.

Instead of elemental sulfur, Li_2_S, the fully lithiated state of sulfur, has also been investigated as the cathode material for next‐generation rechargeable batteries. Utilization of Li_2_S cathode possesses multiple advantages, such as a lower density than elemental sulfur, the volume expansion during cycling can be alleviated effectively. And, Li metal anode can be replaced with graphite, tin or silicon, etc. Moreover, Li_2_S can be easily encapsulated into a complex structured sulfur host via calcination and solution infiltration method because of its high melting point (938 ℃) and high solubility in ethanol. Hence, a milder shuttle effect can be observed compared to traditional elemental sulfur during cycling. Cui and co‐workers designed a 3D graphene cage with a thin layer of electrodeposited nickel phosphosulfides (denoted as Ni‐P‐S) for Li_2_S impregnation.^[^ ^110^ ^]^ The carbon‐based coating can improve the electric conductivity and physically block soluble polysulfides, and the highly conductive and sulfiphilic structure of Ni‐P‐S@G cages enables facile charge transfer and low polysulfides loss during cycling. The resulting Li_2_S cathode delivers a high specific capacity of 543 mAh g^−1^ at 4 C and retains 540 mAh g^−1^ after 300 cycles at 0.5 C.

### 3.2 Tailored Electrolyte Systems

When the adsorption ability of cathode hosts is not enough to anchor the LiPSs, the LiPSs will break away from the cathode surface and migrate to the anode side driven by the concentration gradient. Thus, preventing the dissolution of polysulfide into electrolytes or avoiding the generation of LiPSs with novel electrolyte systems is the second step to prevent the shuttle effect. In this regard, solid‐state electrolytes have also been recognized as an alternative strategy for LiPSs shuttle inhibition. In the following part, the related improvement strategies about liquid electrolytes and solid‐state electrolytes are discussed systematically. **Table** **3** exhibits the detailed information of Li–S batteries fabricated with the tailored electrolytes systems.

**Table 3:** Electrochemical performances of Li–S batteries fabricated with the tailored electrolytes systems

Electrolytes	Cathode material	Mass loading [mg cm^−2^]	Initial capacity [mAh g^‐1^]/C rate	Final capacity [mAh g^‐1^]/cycle numbers	Capacity retention	Refs.
Liquid electrolytes						
1 m LiTFSI/FDE/DOL/DME	KB+S	1.0	1310/0.5 C	701/200	53.5	^[^ ^113^ ^]^
1 m LiFSI/OFE/DME	C+S	1–2	1380/0.05 C	775/150	56.2	^[^ ^114^ ^]^
1 m LiTFSI/DMDS/DOL/DME	C+S	1	1350/0.33 C	1200/250	92.3	^[^ ^116^ ^]^
7 m LiTFSI/DME/DOL	C+S	–	1041/0.2 C	770/100	73.9	^[^ ^118^ ^]^
5 m LiTFSI/DME/DMTS	HPC+C	1.5–2	1400/0.1 C	910/50	65.0	^[^ ^119^ ^]^
Solid‐state electrolytes						
Li_1+_ *_x_* Y*_x_* Zr_2−_ *_x_* (PO_4_)_3_	CNF+Li_2_S_6_	2.0	950/0.2 C	850/150	89.5	^[^ ^120^ ^]^
Li_1.5_Al_0.5_Ge_1.5_(PO_4_)_3_	KB+S	–	1386/0.2 C	720/40	51.9	^[^ ^121^ ^]^
Li_1+_ *_x_*_+_ *_y_* Al*_x_* Ti_2−_ *_x_* Si*_y_* P_3−_ *_y_* O_12_	Carbon+Li_2_S	1.4	–/0.05 C	900/150	–	^[^ ^122^ ^]^
PEO‐LiTFSI‐Al_2_O_3_	–	1.54	1306/0.2 C	680/200	52.1	^[^ ^123^ ^]^
PVDF‐LiTFSi‐polysiloxane	MWCNT+S	2‐3	1493/1 C	828/80	55.5	^[^ ^124^ ^]^
PEO‐LiTFSI‐MIL‐53(Al)	PANI+C+S	0.8	640/0.2 C	558/1000	87.0	^[^ ^125^ ^]^
PEO‐LiTFSI‐Al_2_Si_2_O_5_(OH)_4_	PANI+C+S	–	1350/0.1 C	745/100	55.2	^[^ ^126^ ^]^
PEO‐LiTFSI‐LATP‐Al_2_O_3_	C+S	0.6–1	1035/0.1 C	823/100	79.5	^[^ ^127^ ^]^
PEO‐liClO_4_‐ Li_7_P_3_S_11_	Graphene+S	–	776/0.05 C	393/60	47.4	^[^ ^128^ ^]^

#### 3.2.1 Liquid Electrolytes

The LiPSs are hardly to dissolve in carbonate esters while the highly reactive polysulfides can react with carbonate esters via a nucleophilic addition or substitution reaction, which finally leads to the loss of active sulfur.^[^ ^111^ ^]^ Hence, ether‐based electrolytes are commonly used in Li–S batteries, in which LiTFSI acts as Li^+^ ion source and DOL/DME works as a solvent for LiTFSI. In order to suppress the dissolution of polysulfide into electrolytes, strategies such as preventing the dissolution of LiPSs, changing the reaction pathway of LiPSs, and regulating the concentration of the electrolyte have been proposed.

#### Reducing the Solubility of LiPSs

The polysulfide solubility decreases greatly after fluorination. The reason is that the donor‐ability of the electrolyte weakens greatly with the hindrance of the oxygen afforded by fluorine.^[^ ^112^ ^]^ Wen et al. demonstrated the insolubility of polysulfides by UV–Vis spectra in the optimized fluorinated diether 1,3‐(1,1,2,2‐tetrafluoroethoxy) propane (FDE) electrolyte.^[^ ^113^ ^]^ In this electrolyte, most oxygen atoms from electrolyte liquid can coordinate with Li^+^. Thus, there is no extra oxygen to dissolve LiPSs. As a result, the battery delivers a specific capacity of 701 mAh g^–1^ and more than 99% average Coulombic efficiency after 200 cycles at 0.5C in the optimized electrolyte. Besides, Wang et al. minimized the solubility of LiPSs by adding inert fluoroalkyl ether of 1H,1H,5H‐octafluoropentyl‐1,1,2,2‐tetrafluoroethyl ether (OFE) into LiFSI/DME electrolyte.^[^ ^114^ ^]^ The assembled batteries with OFE into LiFSI/DME electrolyte exhibit superior performance. Furthermore, the fluorinated ethers solvents could also prevent the LiPSs shuttle by forming LiF‐rich SEI film in Li metal surface.^[^ ^115^ ^]^

#### Changing the Reaction Pathway

The shuttle effect is caused by the dissolution and migration of LiPSs in ether‐based electrolytes. Taking in another way, changing the reaction pathway of sulfur to avoid the formation of long‐chain LiPSs is an effective approach to eliminate the shuttle behavior. Wang et al. incorporated dimethyl disulfide (DMDS) into 1 m LiTFSI in DOL/DME to change the sulfur reaction pathway.^[^ ^116^ ^]^ With the increase of DMDS content, the high voltage discharge plateau at 2.4–2.3 V corresponding to the reduction of solid S_8_ to soluble polysulfides was disappeared, and the color change of electrolyte indicates that there was no soluble polysulfides formation during discharge process. Moreover, the operando proton nuclear magnetic resonance analysis verifies that the solid sulfur was reduced as soluble dimethyl polysulfide species to form lithium organosulfides and Li_2_S. Differently, Goodenough et al. reported that bis(4‐nitrophenyl) carbonate (BNC) as highly polar additive in the electrolyte would react with soluble polysulfides to form an insoluble sulfide complex and lithium 4‐nitrophenolate, and then prevent the shuttle effect.^[^ ^117^ ^]^ Profiting from the shuttle‐free feature, the sulfur cathode with BNC‐added electrolyte shows a very high Coulombic efficiency (≈100%) at a loading of 1.4 mg cm ^−2^.

#### Regulating the Electrolytes Concentration

The concentration of the commonly used electrolyte is about 1–1.5 m, which possesses large amounts of free solvent to dissolve LiPSs. While as the concentration increases, free solvent decreases gradually due to contact ion pairs or cation‐anion aggregates solvated sheath. Chen et al. reported a “Solvent‐in‐Salt” electrolyte with ultrahigh salt concentration (7 m), in which case the soluble intermediate became hardly soluble because it has a certain saturation degree for solvent, as proven by LiPSs dissolution experiments.^[^ ^118^ ^]^ Consequently, a Coulombic efficiency nearing 100% and long cycling stability were achieved. However, the active sulfur cathodes still undergo a solid‐liquid‐solid lithiation process and the formation mechanism of LiPSs in high concentration electrolyte is still ambiguous. While the question of where is the free solvent from in concentrated electrolytes has remained. Amine et al. discovered that the solvating power difference among various cations/anions with the solvents could initiate a solvation‐ion‐exchange and then result in solvation and re‐formation of soluble LiPSs. Thus, a concentrated siloxane‐based (DMTS) electrolyte was designed for Li–S batteries, which could effectively eliminate the hidden solvation‐ion‐exchange process and thereby limit the dissolution of LiPSs simultaneously. Correspondingly, the shuttle phenomenon can be suppressed notably.^[^ ^119^ ^]^ Although the concentrated electrolytes have a significant effect on preventing the shuttle effect, the ionic conductivity, viscosity, and cost further need to be improved for the practical application of Li–S batteries.

#### 3.2.2 Solid‐State Electrolytes

**Figure 10:**
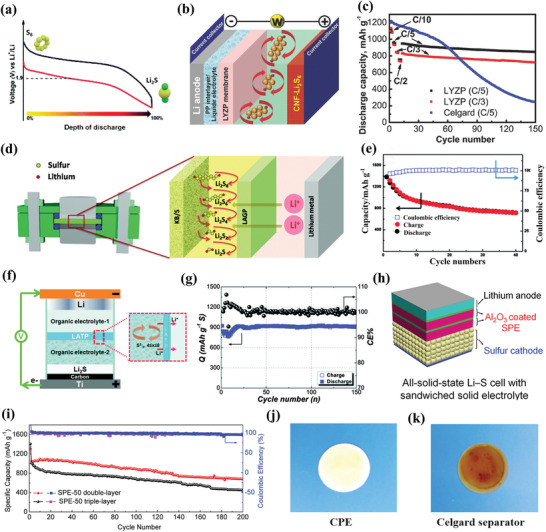
a) Schematic illustration of charge–discharge curves in Li–S batteries with solid‐state electrolyte. b,c) Schematic illustration and cycling performance of Li//LYZP//Li_2_S_6_ cell with a Li metal anode, and a dissolved lithium‐polysulfide cathode. Reproduced with permission.^[^ ^120^ ^]^ Copyright 2016, Wiley‐VCH. d,e) Schematic illustration and cycling performance of Li–S batteries with LAGP SSE. Reproduced with permission.^[^ ^121^ ^]^ Copyright 2014, Royal Society of Chemistry. f,g) Schematic illustration and cycling performance of the Li–S batteries with dual‐phase electrolyte and LATP SSE. Reproduced with permission.^[^ ^122^ ^]^ Copyright 2014, Royal Society of Chemistry. h,i) Schematic illustration and cycling performance of the Li–S batteries with Al_2_O_3_ coating SPE. Reproduced with permission.^[^ ^123^ ^]^ Copyright 2019, Wiley‐VCH. Optical photographs of j) SPE and k) liquid electrolyte + Celgard separator after 80 cycles at 1 C and 25 °C, corresponding to the side contact with the Li anode. Reproduced with permission.^[^ ^124^ ^]^ Copyright 2018, Elsevier.

As described above, great progress in blocking the LiPSs shuttle in cathodes and liquid electrolytes has been achieved. However, the shuttle phenomenon is inevitable in the existence of liquid electrolytes. Besides, the traditional liquid electrolytes used in Li–S batteries contain organic solvents that are usually flammable, corrosive, and thermally unstable, which could cause fire and explosion risks. Replacing the liquid electrolytes with solid‐state electrolytes (SSEs), working as the nonflammable physical barriers to restrain the transfer of LiPSs, is considered as another effective method to alleviate the above issues. As displayed in **Figure** **10**a, Li–S batteries only undergo a one‐step solid–solid reaction in inorganic solid electrolytes, which is different from the liquid system without the formation of the soluble LiPSs intermediates.^[^ ^129^ ^]^ SSEs are mainly classified into inorganic solid electrolytes, polymer electrolytes (SPEs), and composite solid electrolytes (CSEs).

#### Inorganic Solid Electrolytes

Inorganic solid electrolytes have a high Li^+^ transference number, nonflammability, and outstanding thermal stability, which are employed as a physical barrier to restrain the LiPSs shuttle. For rigid oxide‐based electrolytes, the poor interfacial contact with electrode materials limits their application solely. Oxide‐based electrolytes are usually combined with liquid electrolytes or SPEs to improve interfacial contact and decrease interfacial resistance. Although the liquid electrolyte or SPEs would cause slight LiPSs dissolution, the oxide‐based electrolyte still acts as a physical barrier to prevent the LiPSs shuttle. Yu and co‐workers reported a polysulfide‐shuttle‐free Li–S battery with Li_1+_ *_x_* Y*_x_* Zr_2−_ *_x_* (PO_4_)_3_ (*x* = 0−0.15) (LYZP) Li^+^ ion conductive SSE.^[^ ^120^ ^]^ As shown in Figure 10b, the PP membrane presoaked with liquid electrolyte was placed between the LYZP separator and anode. The LiPSs dissolution in liquid electrolyte accelerate the Li^+^ ion diffusion and the electrochemical reaction kinetics, while the LYZP SSE blocked the LiPSs shuttle. As a result, the assembled Li–S batteries retain 90% of the initial capacity after 150 cycles (Figure 10c). Similarly, Wang et al. exploited Li_1.5_Al_0.5_Ge_1.5_(PO_4_)_3_ (LAGP) integrated with LiTFSI/DOL/DME electrolyte (Figure 10d).^[^ ^121^ ^]^ The LAGP surface close to the anode side shows no sulfur appearing, while sulfur was detected on the surface next to the cathode side in the EDS spectrum. And the XPS spectra of Li anode cycled in liquid electrolyte show a much stronger peak intensity of the Li_2_S compared to that cycled in LAGP electrolyte. XPS and EDS results demonstrate that the shuttle effect has been eliminated effectively by LAGP SSE. Thus, the Coulombic efficiency of assembled Li–S batteries was near 100% and the reversible specific capacity is remained 720 mAh g^−1^ after 40 cycles at 0.2 C rate (Figure 10e). Xia and co‐workers reported dual‐phase electrolyte separated by LATP, as shown in Figure 10f.^[^ ^122^ ^]^ The LATP was an ionic conductor which is permeable to Li^+^ ions while impermeable to LiPSs. LiPSs were restrained on the cathode side and the parasitic reactions caused by the polysulfide shuttle were totally eliminated. As a result, the assembled Li–S batteries exhibit 900 mAh g^−1^ of reversible capacity as well as a 100% of Coulombic efficiency over 100 cycles (Figure 10g). In summary, although a small amount of liquid electrolyte used in oxide‐based SSEs system will cause the formation of LiPSs intermediates, oxide‐based SSEs with dense structure and high Young's modulus will work as a physical barrier to prevent LiPSs shuttle effectively.

Sulfide‐based electrolytes possess distinctly higher ion conductivity due to the more polarizable electron cloud of sulfur compared to oxygen. In fact, the ionic conductivity of some sulfide‐based electrolytes is comparable with liquid electrolytes (around 10^−2^ S cm^−1^). For example, Kamaya et al. reported a new 3D framework structured Li_10_GeP_2_S_12_ (LGPS) with high Li^+^ ionic conductivity of 12 mS cm^−1^ at room temperature.^[^ ^130^ ^]^ Due to their softness and processability, sulfide‐based SSEs can be used in Li–S batteries by single‐component without the addition of liquid electrolyte, and the polysulfides shuttle issue can be eliminated due to no formation of soluble LiPSs intermediates. Thus, sulfide‐based electrolytes are the real all‐solid‐state electrolyte. And the assembled batteries with sulfide‐based SSEs are actually shuttle‐free Li–S batteries.

Thio‐LISICON SSEs with the general formula of Li_11−_ *_x_* M_2−_ *_x_* P_1+_ *_x_* S_12_ (M = Ge and Sn) show the highest ionic conductivity at room temperature among sulfur‐based electrolytes. While the M^4+^ metal ions show instability against Li anode, a stable interlayer is needed between Li metal and such SSEs.^[^ ^131^ ^]^ Wang et al. infused LITFSI and LiNO_3_ into glass fiber to produce solid‐state plastic crystal electrolytes (PCEs).^[^ ^132^ ^]^ The PCEs could work as an interlayer to suppress the reaction between Li anode and sulfide‐based SSEs. The discharge‐charge profiles of assembled Li–S batteries show a single plateau, indicating no polysulfides production. And the Li–S batteries exhibit a high initial capacity of 1682 mAh g^−1^ and a low decay rate (0.14% compared to second discharge capacity) after 100 cycles. The ionic conductivity of Li_2_S–P_2_S_5_ glass‐ceramic SSEs is 10^–5^‐10^–3^ S cm^−1^ at room temperature. Similarly, the Li–S batteries with Li_2_S–P_2_S_5_ glass‐ceramic SSEs show single plateau, indicating direct solid‐solid reactions (S_8_ ⇌ Li_2_S). In brief, polysulfide shuttle can be effectively inhibited in all‐solid‐state Li–S batteries based on sulfide‐based electrolytes because of the lack of liquid solvent to generate LiPSs. The direct solid‐solid reactions (S_8_ ⇌ Li_2_S) will occur during charge and discharge processes. However, the narrow voltage window and high reaction activity to moisture and oxygen hinder their practical application in all‐solid‐state Li–S batteries.

#### Solid Polymer Electrolytes (SPEs)

SPEs are commonly fabricated through the dissolution of Li salt into polymer matrice. Poly (ethylene oxide) (PEO) is the most used polymer matrix and LiN(SO_2_CF_3_)_2_ (LiTFSI), Li[N(SO_2_F)_2_] (LiFSI), LiC(CN)_3_ (LiTCM), and LiClO_4_ are common used Li salts. The transport mechanism of Li^+^ ion in PEO SPE is hopping from one coordinating site to another upon segmental motion of the polymer chains.^[^ ^133^ ^]^ Compared to inorganic SSEs, good contact with electrodes, low density, and high flexibility of SPEs are favorable to obtain high gravimetric energy density. However, the ionic conductivity of PEO SPE is relatively low due to the high crystallinity of polymer with high molecular weight at room temperature, which results in the high operating temperature of 60 ℃. Besides, similar to the liquid electrolyte, LiPSs are soluble in the polymer matrix and the elevated operating temperature would exacerbate this phenomenon, leading to the shuttle effect.^[^ ^134^ ^]^

The coating on the SPEs surface can suppress LiPSs shuttle.^[^ ^123^ ^]^ Fan et al. reported a strategy to solve the shuttle effect through coating Al_2_O_3_ on PEO/LiTFSi SPE surface by atomic layer deposition (ALD).^[^ ^123^ ^]^ The Al_2_O_3_ layer not only showed efficient polysulfides adsorption ability but also could alleviate the side reaction between polysulfides and Li anode. The Al_2_O_3_ layer was increased to 50 layers, the resulting cell exhibits a high discharge specific capacity of 1307 mAh g^−1^ and maintains 1080 mAh g^−1^ after 25 cycles (Figure 10h,i). While the cell without Al_2_O_3_ coating could not cycle successfully even in the first few cycles due to the dissolution and migration of LiPSs. Gao et al. fabricated a thin polydopamine (PDA) layer by self‐polymerizing on the surface of PVDF‐based electrolyte. The pyrrolic nitrogen in the PDA structure is beneficial to confine polysulfides through their strong interaction, thus reducing the polysulfide side reaction with the anode. As a result, the assembled battery with such electrolyte shows a capacity of 868.8 mAh g^–1^ with a low capacity decay of 0.14% per cycle after 200 cycles.^[^ ^135^ ^]^

Grafting, copolymerization, and blending are the main ways of introducing functional groups, which can increase the polarity of the polymer molecular chain to enhance the adsorption ability toward LiPSs. Chen et al. fabricated bi‐grafted polysiloxane copolymer by a hydrosilylation reaction of Si–H with C═C under argon atmosphere and dissolved LiTFSI, PVDF into DMF to fabricate SPE.^[^ ^124^ ^]^ The ionic conductivity of the fabricated SPE reached 7.8 × 10^–4^ S cm^−1^ at 25 ℃. And the slight color change was observed at the side of SPE contacting with Li anode, while obvious change appeared on the surface of the Celgard separator after 80 cycles (Figure 10j,k), suggesting that the shuttle behavior of the LiPSs has been effectively blocked. Thus, the assembled Li–S batteries show excellent electrochemical performance at ambient temperature. Wu et al. integrated the PVDF‐based electrolyte with poly(methyl methacrylate) (PMMA). The —O—C═O functional groups in PMMA can trap the dissolved LiPSs to improve cycling stability.^[^ ^136^ ^]^ Similar conclusions have been also demonstrated by Zhao et al.^[^ ^137^ ^]^

In addition, different polymer frameworks with polarity functional groups have been synthesized and applied in solid polymer electrolytes such as poly (methyl methacrylate) (PMMA),^[^ ^138^ ^]^ poly(vinylidene fluoride) (PVDF),^[^ ^139^ ^]^ poly(vinylidene fluoride‐co‐hexafluoropropylene) (PVDF‐HFP),^[^ ^140^ ^]^ and so on. These polymers possess polarity functional groups such as —C═O, —C—O—, C—F, which will increase the adsorption ability of SPEs to LiPSs. Thus, the shuttle effect of LiPSs is effectively blocked.

#### Composite Solid Electrolytes (CSEs)

CSEs are fabricated by adding inorganic fillers into SPEs. Inorganic fillers contain non‐ionically conductors and fast ionic conductors. Oxide, sulfide, and MOFs fillers are widely used in CPE‐based Li—S batteries, which could relieve the shuttle effect by chemisorption between the lone pair electrons of O or S atom and Li atom in the LiPSs. Besides, the added fillers could decrease the crystalline, improve ionic conductivity at room temperature and enhance the mechanical strength of SPEs.

As for non‐ionically conductive fillers, Zhang et al. fabricated MIL‐53(Al) MOF modified PEO SPE which was used to inhibit polysulfide dissolution and shuttle of PANI@C/S cathode by means of the chemical adsorption of the amine groups and strong Lewis acid properties of MIL‐53(Al) MOF.^[^ ^125^ ^]^ The batteries deliver a stable capacity of 876 mAh g^−1^ at 80 ℃ under a current density of 0.2 C with excellent cycling stability. Lin et al. reported PEO‐based SPE decorated with Al_2_Si_2_O_5_(OH)_4_ nanotube, which has negatively charged outer silica surface and positively charged inner aluminum group surface.^[^ ^126^ ^]^ This structure could promote LiTFSI dissociation, facilitate Li^+^ transport and suppress LiPSs shuttle. The ionic conductivity of 1.11 ×10^–4^ S cm^−1^ was obtained at 25 ℃. The assembled Li—S batteries present a stable discharge capacity of 745 mAh g^−1^ after 100 cycles with a capacity retention of 87%. And the Coulombic efficiency was close to 100%, indicating that the shuttle effect of LiPSs was inhibited greatly. Li et al. reported a bilayer PEO electrolyte via adding Al_2_O_3_ or Li^+^ ion conductive glass‐ceramic as fillers.^[^ ^141^ ^]^ The high surface energy of Al_2_O_3_ could absorb soluble polysulfides while the Li^+^ ion conductive glass‐ceramic work as a physical barrier to block LiPSs shuttle. With the synergistic effect of these two layers in preventing LiPSs shuttle, the Coulombic efficiency of Li—S batteries was improved to 99%.

The fast ionic conductor has high ionic conductivity (above 10^−4^ S cm^−1^) at room temperature. However, they show poor contact properties with electrodes. Combining fast ionic conductors with SPEs with good electrodes compatibility is a promising strategy to improve contact performance and then decrease interface resistance. Liang et al. designed a PEO/LATP composite solid electrolyte coated by Al_2_O_3_/PEO sandwich structure.^[^ ^127^ ^]^ the dissolved LiPSs in PEO SPE could be blocked by LATP inorganic electrolyte. The Al_2_O_3_ layer via ALD coating further prevents the reduction of LATP by polysulfide species. As a result, the Li–S batteries with sandwich‐structured composite solid electrolyte show a stable cycling performance with a discharge capacity of 823 mAh g^−1^ after 100 cycles at 0.1 C, which was two times higher than that with a liquid electrolyte. Xu et al. synthesized Li_7_P_3_S_11_/PEO CSE.^[^ ^128^ ^]^ PEO–LiClO_4_ not only works as a conductive bridge between Li_7_P_3_S_11_ particles but also could decrease the electrolyte interfacial resistance successfully, which increases the ionic conductivity from 1.4 ×10^–3^ to 2.1 ×10^–3^ S cm^−1^ at room temperature. And the dissolved LiPSs in PEO would be inhibited by Li_7_P_3_S_11_ according to only one discharge plateau. Thus, the assembled Li–S batteries exhibited greatly improved cycling stability and higher electrochemical reaction reversibility.

In summary, in the inorganic all‐solid‐state electrolyte, oxide‐based electrolytes are usually combined with liquid electrolytes or polymer electrolytes to improve interfacial contact and decrease interfacial resistance, which results in an unavoidable polysulfide shuttle. And similar phenomenon exists in solid polymer electrolytes, the LiPSs can dissolve in the polymer matrix to generate the shuttle effect. Coating some functional materials on the SPEs surface or introducing polarity groups (—O—C═O, —C═O, —C—O—C, —C═N), strong Lewis acid groups and inorganic fast ionic conductor can adsorb or physical shielding LiPSs and then relieveshuttle behaviors. In a word, sulfide‐based solid electrolytes or composite solid electrolytes without polymers/liquid components would be the best choices to achieve the shuttle free Li–S batteries.

### 3.3 Functional Separators

In the liquid electrolyte, the dissolution of LiPSs is unavoidable. When the electrolyte regulating strategies cannot completely prevent the dissolution and diffusion of LiPSs, the LiPSs will diffuse to the separator's interface driven by the concentration gradient. As the third step to suppress the shuttle effect of LiPS, the function of separators is equivalent to a fence that prevents the passage of LiPSs. The average size of the long‐chain LiPSs is several nanometers, while the pore size of routine commercial separators such as polypropylene porous membrane (PP) or polyethylene porous membrane (PE) is up to 100 nm. Hence, the soluble LiPSs can easily penetrate the separators to the anode side.^[^ ^142^ ^]^ In this regard, functional separators with physical shielding effects and chemical trapping effects that can block soluble LiPSs or catalyze their conversion have been intensively studied to impede the diffusion behavior. **Table** **4** summarizes the electrochemical performances of Li–S batteries fabricated with modified separators.

**Table 4:** Electrochemical performances of Li–S batteries fabricated with functional separators

Modified separators	Cathode materials	Coating loading [mg cm^−2^]	Coating thickness [µm]	Initial specific capacity/C rate	Final capacity/cycle number/C rate	Capacity retention/decay rate [%]	Refs.
Physical shielding effect							
MPC/PEG	S/C	0.15	8	1307/0.2 C	596/500/0.2 C	0.108/0.2 C	^[^ ^143^ ^]^
MWCNT/PP	S/Super P	0.17	–	1324/0.2 C	624/600/1 C	0.14/1 C	^[^ ^145^ ^]^
CVD‐2G/PP	S/Ketjen Black	0.15	6	1460/0.1 C	600/1500/0.5 C	0.026/0.5 C	^[^ ^150^ ^]^
Super P/PP	S/Super P	0.2	20	1389/0.2 C	701/200/0.2 C	0.16/2 C	^[^ ^151^ ^]^
CFs/PP	S/Super P	0.14	40	1063/0.5 C	683/500/0.5 C	0.071/0.5 C	^[^ ^146^ ^]^
Nafion‐PP/PE/PP	CNT–S	0.7	1	781/1 C	468/500/1 C	0.08/1 C	^[^ ^147^ ^]^
Chemical trapping effect							
rGO@PP/SL	S/C	0.2	–	707/2 C	523/1000/1 C	0.026/2 C	^[^ ^148^ ^]^
S_6_^2−^‐VPP	S/C	–	2	1310/0.1 C	578/2000/3 C	0.012/3 C	^[^ ^149^ ^]^
G@PC/PP	CNT/S	0.075	0.9	1476/0.2 C	754/500/1 C	0.023/1 C	^[^ ^152^ ^]^
Oxi‐d‐MXene600/PP	S/oxi‐d MXene900	9.3	119	1408/1 C	800/300/1 C	0.001/1 C	^[^ ^153^ ^]^
Cu_2_(CuTCPP)/PP	C/S	0.1	0.5	850/1 C	604/900/1 C	0.032/1 C	^[^ ^154^ ^]^
MoO_3_/PP	CNT_S_/S	0.45	–	1377/0.5 C	782/200/0.5 C	0.002/0.5 C	^[^ ^155^ ^]^
ZnS@WCF/PP	C/S	–	12.6	1029/0.5 C	685/600/1 C	0.045/1 C	^[^ ^156^ ^]^
LDH@NG/PP	S/C	0.3	1.5	812/2 C	337/1000/2 C	0.06/2 C	^[^ ^157^ ^]^
VN/PP	S/C	–	25	1050/0.5 C	385/800/1 C	0.077/1 C	^[^ ^158^ ^]^

#### 3.3.1 Physical Shielding Effect

The soluble LiPSs shuttle phenomenon can be suppressed efficiently by trapping them via customized separators with a physical shielding effect. To be specific, the physical shielding effect refers to that long‐chain LiPSs are anchored into the coating materials of the modified layer through physical absorption or repelled to the cathode side through electrostatic repulsion.

#### Physical Absorption

**Figure 11:**
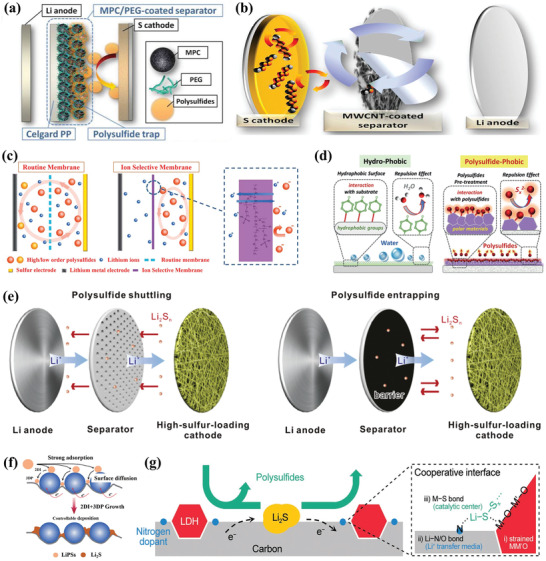
a) Schematic illustration of a Li–S cell with MPC/PEG‐coated separator. Reproduced with permission.^[^ ^143^ ^]^ Copyright 2014, Wiley‐VCH. b) The Li–S cell with an MWCNT‐coated separator. Reproduced with permission.^[^ ^145^ ^]^ Copyright 2014, American Chemical Society. c) Schematic illustration of the Li–S batteries with routine and ion‐selective separators. Reproduced with permission.^[^ ^147^ ^]^ Copyright 2014, Royal Society of Chemistry. d) Schematic illustrations of hydrophobic surface and polysulfide‐phobic surface. Reproduced with permission.^[^ ^149^ ^]^ Copyright 2019, Wiley‐VCH. e) Shuttle behavior of LiPSs on conventional PP and G@PC/PP Separators. Reproduced with permission.^[^ ^152^ ^]^ Copyright 2018, CellPress. f) Schematic illustration of the Li_2_S deposition on ZnS@WCF. Reproduced with permission.^[^ ^156^ ^]^ Copyright 2020, Royal Society of Chemistry. g) Schematic illustration of the cooperative interface of LDH@NG. Reproduced with permission.^[^ ^157^ ^]^ Copyright 2016, Wiley‐VCH.

Similar to the sulfur host materials mentioned above, the physical absorption of separators is closely related to the van der Waals force. It is worth noticing that LiPSs would not be entrapped well, or the absorbed LiPSs will separate away from the adsorbent easily if the adsorption is too weak. The modified materials with physical absorption effect are mainly relied on their high porosity and surface areas such as porous carbon,^[^ ^143^ ^]^ carbon black,^[^ ^144^ ^]^ and carbon nanotubes.^[^ ^145^ ^]^ A composite separator with polyethylene glycol (PEG)‐supported microporous carbon (MPC) coating on PP separator was proposed by Manthiram's group.^[^ ^143^ ^]^ The conductive/porous MPC/PEG coating serves as a polysulfides trap for suppressing polysulfides diffusion (**Figure** **11**a). After cycling, the specific surface area of the MPC/PEG coating is decreased to 49 m^2^ g^−1^, which is much lower than the initial value of 1321 m^2^ g^−1^. Moreover, the scraped surface of the cycled MPC/PEG‐coated separator did not show the elemental sulfur signals, demonstrating the excellent LiPSs‐trapping capability of the MPC/PEG coating.

Besides, a multiwalled CNT (MWCNT)‐modified separator was synthesized by coating the MWCNTs as a porous filter on a conventional porous separator.^[^ ^145^ ^]^ Profiting from the high conductivity and ultralight weight properties of MWCNTs, this modified separator could provide several enhancements for Li–S system such as the efficient entrapment of the free migration of LiPSs, the reactivation of the trapped sulfur species, and high mass‐energy density for practical application (Figure 11b). In addition to porous carbon and carbon nanotubes, Zheng et al. have reported ultralight carbon flakes modified separator (CFs@PP).^[^ ^146^ ^]^ Analogously, this carbon layer coating works as an effective LiPSs barrier and the assembled cell delivers an initial discharge capacity of 1063 mAh g^−1^ and retains 683 mAh g^−1^ after 500 cycles at 0.5 C. Overall, these carbonous modified layers can not only act as an airtight shield for preventing the soluble LiPSs from diffusion but also work as a second current collector and facilitate electron transportation, reactivating the trapped active material and promoting the sulfur utilization.

#### Electrostatic Repulsion

The electrostatic repulsion force exists in the two molecules with the same charge, which can be utilized to design the modified materials that provide strong obstruction against LiPSs. To be specific, the S*_x_* ^2−^ ions in long‐chain LiPSs exhibit a negative charge, so they will be obstructed by the ions or groups with the same negative charge while guaranteeing the transportation of positively charged Li^+^ ions.^[^ ^147^ ^]^ Huang and co‐workers reported an ion‐selective strategy by introducing sulfonate‐ended perfluoroalkyl ether groups on the ionic separators.^[^ ^147^ ^]^ The —SO^3−^— groups exhibit a strong negative charge effect, hence, these —SO^3−^— groups allow ion hopping of positively charged species (Li^+^ ion) but reject hopping of negative ions (S*_x_* ^2−^) due to the Coulombic interactions (Figure 11c). Consequently, the prepared cell with this customized membrane delivers a low decay rate of 0.08% per cycle after 500 cycles. Likewise, a graphene composite separator was prepared with abundant sulfonic groups for rejecting the soluble LiPSs.^[^ ^148^ ^]^ This composite separator was synthesized by polymerizing hexamethylene diisocyanate with reduced graphene oxides and sodium lignosulfonates (RGO@SL/PP) directly. The rich negative charge in the lignin porous network could suppress the translocation of the negatively charged polysulfides ions while permitting the transportation of Li^+^ ions. Additionally, inspired by the repulsion effect of the hydrophobic surface towards H_2_O molecules, He et al. proposed the “polysulfide‐phobic” strategy by introducing the VOPO_4_ sheets onto the commercial separator.^[^ ^149^ ^]^ In this system, a portion of the soluble LiPSs was firstly anchored on the separator by polar VOPO_4_ through the formation of a V—S bond and the surface anchored with polysulfides can evolve into a polysulfide–phobic interface, which spontaneously rejects the diffusion and shuttle of the dissolved polysulfide anions (Figure 11d). Furthermore, this strategy is not limited to VOPO_4_ but can be further extended to other polar materials, which can easily adsorb polysulfide, resulting in the repulsively interaction with free polysulfide in bulk solution.

#### 3.3.2 Chemical Trapping Effect

The physical shielding effect is far from meeting the practical requirements of Li–S batteries. In other words, the dissolution and diffusion of LiPSs cannot be eliminated fundamentally based on the idea of blocking. To improve the overall obstruction effect, chemical trapping effects have been also introduced to separators. Chemical trapping behaviors are divided into two categories: chemical adsorption and electrochemical catalysis.

#### Chemical Adsorption

Similar to sulfur hosts, chemical adsorption refers to the fact that the coating materials could immobilize LiPSs by electron transfer, exchange, or chemical bonding effect, which is much stronger than the van der Waals force. Thus, the as‐absorbed LiPSs will not easily fall off the coating materials with polar–polar interactions or Lewis acid–base interactions.

Analogously, heteroatom doping has been widely used to enhance the polarity of nonpolar carbon coating materials. Zheng and co‐workers investigated a powerful functional separator by coating 2D nitrogen‐doped porous carbon nanosheets on the commercial PP separator.^[^ ^152^ ^]^ In addition to the complete physical barrier constructed by the close‐packed assembly of 2D nanosheets, the high content of nitrogen dopants and ultrahigh specific surface area offered abundant adsorption sites to capture the LiPSs via chemisorption (Figure 11e). The Li–S batteries with the modified separator exhibit a high discharge capacity of 1186 mAh g^−1^ after 100 cycles at 0.2 C. Manthiram's group proposed two kinds of functional separators with the coating of boron‐ and nitrogen‐doped reduced graphene oxide (B‐rGO, N‐rGO).^[^ ^159^ ^]^ The introduction of heteroatoms into rGO frameworks can increase the chemical affinity toward LiPSs. The cells with heteroatoms modified separator obtained higher reversible capacities and more stable cyclic performance than bare rGO. Apart from the heteroatomic doped carbon materials, metal compounds such as metal sulfides, metal oxides, and metal nitrides can be directly employed as chemosorbents to anchor LiPSs based on their intrinsic polarity.^[^ ^160^ ^]^ The electrocatalytic effect is always along with the adsorption effect simultaneously, we will elaborate on the adsorption of these metal compounds in the following electrocatalysis section.

Since metal ions can accept the extra electrons from LiPSs through Lewis acid–base interactions, MOFs and MXenes are considered promising coating materials for separator modification. Tian et al. have developed a highly oriented MOF membrane by vacuum filtering the few‐molecular‐layer thin copper‐based MOF nanosheets suspension (Cu_2_(CuTCPP)/PP).^[^ ^154^ ^]^ The Cu_2_(CuTCPP) membrane can capture the LiPSs through Lewis acid–base interactions, hence reducing the permeability of LiPSs significantly. The Cu_2_(CuTCPP)‐modified separator obtained a capacity retention rate as high as 71.1% after 900 cycles at 1C with a high sulfur loading of 10 mg cm^−2^. Recently, a CO_2_‐oxidized Ti_3_C_2_T*_x_*‐MXenes component was proposed for sulfur host and separator coating material by Lee et al.^[^ ^153^ ^]^ The CO_2_‐oxidized MXenes consisted of 2D carbide flakes with a rutile‐TiO_2_ crystal structure and the Ti sites show strong chemical affinity toward LiPSs, and the shuttle behavior of soluble LiPs are inhibited effectively by the synergistic effect of physical and chemical adsorption.

#### Electrochemical Catalysis

Tapping the soluble LiPSs by adsorption effects mentioned above can mitigate the shuttle effect to a certain extent. And the adsorption sites will be used up and the passway of Li^+^ might be blocked with the accumulation of LiPSs on the separators, leading to the rapid degradation of battery performances. Therefore, it is necessary to accelerate the conversion of the as‐absorbed sulfur species on the separators. To this end, the electrocatalyst materials used for the separator modification are becoming popular because they cannot only trap the LiPSs but also catalyze the conversion process. For instance, Imtiaz et al. have proved that the MoO_3_ coating layer can not only impede the shuttle behavior of LiPSs but also significantly accelerate the redox conversion reaction of LiPSs through the formation of surficial O*_x_* S*_n_* moieties.^[^ ^155^ ^]^ The resultant Li–S batteries exhibit a very high initial capacity of 1377 mAh g^−1^ and retain 684.4 mAh g^−1^ after 200 cycles at 0.5 C.

Cao and co‐workers designed a novel functional barrier by wrapping dispersed zinc sulfide nanospheres with a graphene‐like ultrathin wrinkled carbon film (ZnS@WCF).^[^ ^156^ ^]^ According to the XPS results, the ZnS exhibited strong chemical interaction toward LiPSs through the combination of S–Zn and Li–S binding. Moreover, ZnS@WCF exhibited the highest nucleation capacity in the Li_2_S nucleation test, demonstrating that ZnS@WCF is favorable to reducing the nucleation barrier and facilitating the liquid‐solid phase transformation (Figure 11f). Benefiting from the excellent catalytic ability, Li–S batteries based on ZnS@WCF modified separator retain a high reversible capacity of 685 mAh g^−1^ after 600 cycles at 1C. Analogously, the modified separator with a cooperative interface by growing the NiFe layered double hydroxide (LDH) nanoplates on the nitrogen‐doped graphene (NG) was constructed.^[^ ^157^ ^]^ The LiPSs could be anchored chemically by the “lithiophilic” NG through the Li—N bond. Fe doping and M–N interaction in “sulfiphilic” LDH can mediate the M–S interaction, thus promoting the conversion of as‐absorbed LiPSs (Figure 11g). Besides, Song et al. used VN nanobelts to modify the commercial separator to block the LiPSs from diffusing.^[^ ^158^ ^]^ VN enables smooth transport channels for lithium ions due to its porous structure, and the polar surface provides effective adsorption sites for LiPSs. Apart from that, other kinds of metal compounds that feature catalytic ability such as metal carbides, metal practicals, and single atoms have been also explored and applied to eliminate the shuttle effect.^[^ ^161^ ^]^

In a nutshell, blocking LiPSs from migration in the liquid electrolyte by functional separators has been proved to be a feasible way to inhibit the shuttle effect. However, a thick or close‐grained modified layer will seriously block the Li^+^ ions transport channel on the original commercial separator, resulting in a reduced Li^+^ ions transport efficiency. Besides, the usage of heavy materials may increase the weight proportion of the modified separator, and then decrease the energy density of batteries to some extent. Hence, the coating materials onto the separators should always meet the following requirements: 1) excellent polysulfide inhibition or adsorption effects, 2) high Li^+^ ions transport efficiency, 3) as thin and lightweight as possible to avoid an evident decrease in energy density, 4) excellent thermal, chemical, and mechanical stability and facile fabrication process.

### 3.4 Anodes Surface Engineering

If the strategies via using modified sulfur hosts, functional separators, and tailored electrolyte composition are insufficient to eliminate the shuttle behavior of LiPSs in the liquid system, in turn, part of the long‐chain soluble LiPSs would diffuse toward the Li anode side inevitably under the concentration gradient. As the end of the soluble LiPSs shuttle path, Li metal anode faces more severe challenges. Once soluble LiPSs shuttle to anode surface via separator and react with Li metal, insoluble and inert Li_2_S and Li_2_S_2_ would be formed, leading to the loss of active sulfur species, irreversible Li consumption, and the degraded Coulombic efficiency. And the subsequently formed inert and heterogeneous SEI layer on the anode surface would hinder Li^+^ ion diffusion, which induces nonuniform current density distribution and subsequently uncontrollable Li dendrite rapid growth, eventually leading to poor cycling performance and safety hazards. This negative effect of LiPSs on Li anode is usually neglected in coin cells with a high N/P ratio (>150) and low sulfur loading.^[^ ^162^ ^]^ However, in practical Li–S pouch cells, the fatal deterioration of Li metal anode is one of the main reasons for rapid failure with dramatic capacity loss.^[^ ^163^ ^]^ The limited Li mass loading in the pouch cell with a low N/P ratio cannot afford continuous irreversible Li consumption caused by reacting with LiPSs. And high LiPSs concentration induced by high sulfur loading causes more severe shuttle and Li corrosion.^[^ ^4b^ ^]^

In fact, if LiPSs can commute between cathode and anode reversibly and avoid reacting with Li anode, Unfortunately, this reaction is spontaneous when Li metal with high reducibility contacts with highly oxidizable LiPSs, especially long‐chain LiPSs. Therefore, it is necessary to prevent the contact and continuous reaction between LiPSs and Li anodes, which could be inhibited by Li anode surface engineering.^[^ ^164^ ^]^

Whereas, to protect Li anodes from the corrosion of soluble LiPSs with anode surface engineering, it is important to regulate the factors affecting the detrimental reaction between Li anode and LiPSs, such as reaction activity of Li anodes and LiPSs, LiPSs concentration, and the exposed surface area of Li anode. The high driving force induced by the increased LiPSs concentration intensifies Li corrosion under practical conditions with high S loading and low N/P ratio. Dramatic volume changes and uneven deposition of Li anode during cycling will amplify the surface area of exposed Li, leading to serious side reactions and the formation of unstable and thickened SEI layer.

To overcome these challenges, exploring effective strategies to engineer Li anode surface is of great importance and significance. Introducing a passivation layer onto the interface is effective. The protection layer needs to inhibit the diffusion of LiPSs and transport Li^+^ ions quickly, as well as prevent irregular Li dendrites from breaking the SEI layer. The strategies based on in situ SEI layers and artificial protection layers have been summarized in the following part. **Table** **5** summarizes the electrochemical performances of Li–S batteries fabricated with Li anode protection strategies.

**Table 5:** Electrochemical performances of Li–S batteries fabricated with Li anode protection strategies

Materials	Cathode material	Mass loading [mg cm^−2^]	Initial capacity [mAh g^‐1^]/C rate	Final capacity [mAh g^‐1^]/cycle numbers	Capacity retention [%]	Refs.
In situ SEI layer						
LiHFDF salt	C/S‐64	9.05	633/0.1 C	424/120	67	^[^ ^189^ ^]^
SOCl_2_ additive	pPAN@S	1.0	1750/0.24 C	1500/200	85.6	^[^ ^182^ ^]^
ZrO(NO_3_)_2_	C/S	1.5	1306/0.5 C	830/280	63.5	^[^ ^173^ ^]^
biphenyl‐4,4′‐dithiol	S+carbon black	0.7–1.5	780/0.1 C	575/300	74	^[^ ^181^ ^]^
Artificial protection layer						
PDMS selective permeable interphase	S+CNT	1.2	1000/0.2 C	800/100	80	^[^ ^192^ ^]^
Hybrid Li_3_Sb/LiF‐based SEI	S+acetylene black	2	1200/0.5 C	900/100	75	^[^ ^193^ ^]^
Lithium 1,3,5‐benzenetrithiolate	S+carbon paper	1.76	1239/1.0 C	907/300	87.6	^[^ ^186^ ^]^
Li*_x_* SiS*_y_*/Nafion	S+CNT	4.5	1140/0.05 C	905/100	79.3	^[^ ^194^ ^]^
A compact inorganic layer	S+CNT	1	900/2 C	725/100	80.6	^[^ ^195^ ^]^
Li_3_Bi/LiF	S+CNF	6.8	1038/0.24 C	942/200	90.7	^[^ ^35^ ^]^

#### 3.4.1 In Situ SEI Layer

The SEI layer formed at the interface plays a decisive role in preventing LiPSs from interacting with Li anode. However, the SEI layer with mosaic structure^[^ ^165^ ^]^ is insufficient to maintain physical and chemical stability when facing to LiPSs with strong corrosivity, which would induce heterogeneous nucleation of Li^+^ ion and uncontrollable Li deposition, in turn deteriorating the SEI layer. And the insoluble Li_2_S and Li_2_S_2_ are formed by the reaction between LiPSs and Li anode and then become the components of SEI layer near Li surface, which would hinder Li^+^ ion transportation.^[^ ^166^ ^]^ Therefore, the main components of the SEI layer, introduced by prior reaction between Li anode with strong oxidants in the electrolyte, play a crucial role in adjusting the subsequent Li stripping/plating behaviors. To optimize the SEI components for protecting Li anode, massive electrolyte additives and solutions have been investigated.

#### Nitrate‐Containing In Situ SEI Layer

In traditional Li–S battery electrolyte systems (DOL/DME with LiTFSI), the thin SEI layer derived from the decomposition of DOL and LiTFSI contains LiOR, HCO_2_Li, Li_2_NSO_2_CF_3_, Li_2_SO_2_CF_3,_ and Li*_x_* CF*_y_*, which fails to block LiPSs shuttle and leads to severe capacity degradation.^[^ ^167^ ^]^ LiNO_3_ with strong oxidation ability as the Li salt was initially introduced by Aurbach et.al. to react preferentially with Li anode in the Li–S electrolyte systems and the Li*_x_* NO*_y_* species would be formed on the surface, making the SEI layer more stable and thicker.^[^ ^168^ ^]^ It was demonstrated that adopting LiNO_3_ as Li salt cannot reduce the dissolution of LiPSs, but could suppress the contact between Li anode and LiPSs by constructing the SEI layer containing Li*_x_* NO*_y_* species.^[^ ^169^ ^]^ The mechanism of the improved electrochemical performance was analyzed by ex‐situ pretreatment through immersing Li metal into LiNO_3_‐containing electrolyte.^[^ ^170^ ^]^ A smooth and compact surface layer, making up of inorganic species such as LiN*_x_* O*_y_* and organic species such as ROLi and ROCO_2_Li, can prevent the parasitic reaction between Li anode and LiPSs. Except for Li^+^ cation, nitrates comprised of other cations were served as additives, such as CsNO_3_,^[^ ^171^ ^]^ KNO_3_,^[^ ^172^ ^]^ and ZrO(NO_3_)_2_,^[^ ^173^ ^]^ where oxidative NO_3_^–^ ion always plays a role in reinforcing SEI layer on the surface of Li metal anode, and the cations have their special functions. For example, Jia et al. verified that further growth of Li dendrites can be delayed by the electrostatically attracted K^+^ cations from the KNO_3_ additive.^[^ ^172^ ^]^

**Figure 12:**
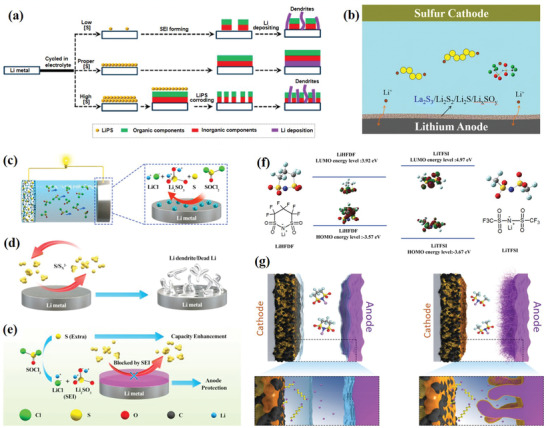
a) The role of LiPS concentrations on SEI layer evolution and Li deposition. Reproduced with permission.^[^ ^176^ ^]^ Copyright 2016, Elsevier. b) Schematic illustration of the formed passive composite film on the surface of Li anode by adding La(NO_3_)_3_ into the electrolyte. Reproduced with permission.^[^ ^185^ ^]^ Copyright 2016, American Chemical Society. c) The reaction between SOCl_2_ and Li anode; d) anode failure without protection; e) anode protection and capacity enhancements by adding SOCl_2_. Reproduced with permission.^[^ ^182^ ^]^ Copyright 2019, Elsevier. f) The molecular structure, molecular model, and HOMO and LUMO energies of LiTFSI and LiHFDF and g) the schematic illustration of how LiHFDF suppresses Li‐dendrite growth and the dissolution/shuttle of LiPSs. Reproduced with permission.^[^ ^189^ ^]^ Copyright 2020, Wiley‐VCH.

Moreover, with the help of LiNO_3_, sulfur species are oxidized into the Li*_x_* SO*_y_* component in the SEI layer,^[^ ^168^ ^]^ which proves the synergy effect between LiPSs and LiNO_3_. Cui's team used Li_2_S_8_ and LiNO_3_ as Li salts and manipulated their concentrations to design a highly stable SEI layer to suppress the growth of Li dendrites.^[^ ^174^ ^]^ Similarly, Li_2_S_5_ with different concentrations was employed as Li salt by Zhang and co‐workers and coupled with LiNO_3_, where an electrolyte containing 0.02 m Li_2_S_5_ (0.10 m sulfur) and 5.0 wt% LiNO_3_ contributed to forming the LiF‐Li_2_S*_x_*‐rich SEI layer to protect the Li anode in common LiTFSI‐based electrolytes (**Figure** **12**a).^[^ ^175^ ^]^ In contrast, a continuous SEI layer is hard to be formed under a low Li_2_S_5_ concentration, where the ample S atoms would interact with the Li atoms at Li (110), which would impede new molecules into the exposed active sites and cause the formation of Li_2_S with distorted (111) planes rather than perfect ones. And Li_2_S_5_ with too high concentration would corrode the SEI layer and Li anode due to its high oxidability.^[^ ^176^ ^]^ Except for LiPSs concentration, LiPSs species also influence the electrochemical stability of the SEI layer and Li deposition behavior. Among different orders of LiPSs (Li_2_S*_x_*, *x* = 1, 2, 3, 4, 5, 6, 7, 8; [S] = 0.1 mol L^−1^), Li_2_S_5_ as Li salt added in LiNO_3_‐containing electrolyte can reap the highest Coulombic efficiency.^[^ ^177^ ^]^

However, NO_3_^–^ ions are consumed continuously during cycling, and an endlessly increasing resistance of the passivation layer is detected in LiNO_3_‐containing catholyte.^[^ ^178^ ^]^ In addition, the strong oxidation ability of NO_3_^–^ puts Li–S batteries at risk of thermal runaway.

#### Sulfide‐Containing In Situ SEI Layer

In common LiTFSI‐based electrolytes, Li–S batteries display a more stable electrolyte/anode interface than that in LiFSI‐based electrolytes because a much reversible LiS*_x_* is formed on the anode rather than inert LiSO*_x_* owing to the difference in bond strength of the salt anions.^[^ ^179^ ^]^ It follows that sulfide components were introduced into the SEI layer to reinforce the interface via different additives (P_2_S_5_,^[^ ^180^ ^]^ biphenyl‐4,4′‐dithiol,^[^ ^181^ ^]^ SOCl_2,_^[^ ^182^ ^]^ etc.) or salts. For instance, Cu_2_S was eventually formed by adding copper powders to capture LiPSs, and a high capacity of 1300 mAh g^−1^ with sulfur utilization of 77.6% was obtained. Similarly, a passivation film mainly containing Li_2_S/Li_2_S_2_/CuS/Cu_2_S was constructed on the surface of Li anode by introducing copper acetate in a polysulfide‐rich environment.^[^ ^183^ ^]^ Transition metal cation nitrates (including Zn^2+^, Cu^2+^, Co^2+^, Ni^2+^, and Mn^2+^) were employed as additives to react with the lower‐order polysulfide anions to form transition metal sulfide particles, which could enhance the stability of the SEI layer and enable a smoother surface with less sulfur, especially Zn^2+^ additive.^[^ ^184^ ^]^ As shown in Figure 12b, La(NO_3_)_3_ additive could also form a composite passivation film including Li_2_S_2_/Li_2_S and Li*_x_* SO*_y_*, as well as Li*_x_* La_2_S_3_ with high ionic conductivity by reacting with LiPSs, which would accelerate Li^+^ ion diffusion and decrease the reducibility of metallic Li. In comparison to other metal cations with strong Lewis acidity, La^3+^ cations with small ion charge/ion radius could not catalyze the ring‐opening polymerization of DOL.^[^ ^185^ ^]^ Besides, a passivation layer which is mainly constituted by highly soluble binary sulfide Li_3_PS_4_ was synthesized via compositing P_2_S_5_ additive with reduction products of Li_2_S and L_2_S_2_. This passivation layer not only alleviates the Li corrosion but also accelerates Li^+^ ion transport.^[^ ^180^ ^]^ By the fair acidity of the sulfhydryl group, organothiol additive, i.e., 1,3,5‐benzenetrithiol (BTT), can react with alkalis to form thiolate salts, i.e., Li_3_‐BTT, which interrupts the adverse reactions and intensifies the Li^+^ ion conductivity. Irregular voids left in the BTT–Li interface layer during exchange reaction were filled by LiF particles from the electrolyte decomposition, forming an even and compact organic/ inorganic protective layer.^[^ ^186^ ^]^ Another additive, thionyl chloride (SOCl_2_), can react with Li anode spontaneously to establish dual‐functions LiCl‐rich surface film (Figure 12c–e). The generated S from the redox reaction provides extra capacity and another product Li_2_SO_3_ contributes to densifying the SEI layer to prohibit the shuttle of LiPSs.^[^ ^182^ ^]^

#### Halide‐Containing In Situ SEI Layer

No only sulfides, halides components also can help to reinforce the in situ SEI layer. E.g. another Li^+^ ion salt, lithium iodide (LiI), was added into the electrolyte without LiPS or LiNO_3_ additives and still enables a high capacity of 1400 mAh g^−1^ at 0.2 C and outstanding cycling stability. It is because the generated I radicals would promote the formation of the comb‐branched polyether protective film on the cathode and the smooth SEI layer with the net‐like porous structure on Li anode, which could suppress LiPSs dissolution on the cathode and reduction on the anode effectively.^[^ ^187^ ^]^ Fluorinated interphases were formed by introducing fluoride‐containing additives, such as lithium oxalyldifluoroborate (LiODFB),^[^ ^188^ ^]^ lithium 1,1,2,2,3,3‐hexafluoropropane−1,3‐disulfonimide (LiHFDF)^[^ ^189^ ^]^) and solvents (such as bis(2,2,2‐trifluoroethyl) ether (BTFE),^[^ ^190^ ^]^ 1,1,2,2‐tetrafluoroethyl‐2,2,3,3‐tetrafluoropropyl ether (TTE).^[^ ^191^ ^]^ As depicted in Figure 12f,g, the introduction of LiHFDF can construct LiF‐rich protective layer on the surface of Li anode and sulfur/carbon cathode, respectively.^[^ ^189^ ^]^ Besides, BTFE cosolvent would react with LiNO_3_ to form a robust SEI layer and shut down the shuttle access of LiPSs to Li anode.^[^ ^190^ ^]^ The in situ formed SEI layers by reacting with LiPSs, additives, solvents and/or Li anode is effective and facile, nevertheless, their components are complicated and uncontrollable. Desired protective layers can be designed and customized reasonably by ex situ methods.

#### 3.4.2 Artificial Protective Layer

An artificial protection layer is expected to barricade physically the permeation of LiPSs or repel LiPSs away from Li anode through distinctive interactions with LiPSs. The customized protection layers need to be chemically and electrochemically stable with electrolytes and LiPSs, and have a strong mechanical modulus against Li dendrites. Dense inorganic and/or organic films with high ionic conductivity are often employed to separate Li anodes from LiPSs and electrolytes physically, such as Al_2_O_3_,^[^ ^196^ ^]^ LiF,^[^ ^197^ ^]^ Li*_x_* SO*_y_*/Li*_x_* NO*_y_*,^[^ ^198^ ^]^ PDMS,^[^ ^199^ ^]^ and Nafions/polyvinylidene difluoride (PVDF).^[^ ^200^ ^]^

#### Inorganic Physical Protective Layer

**Figure 13:**
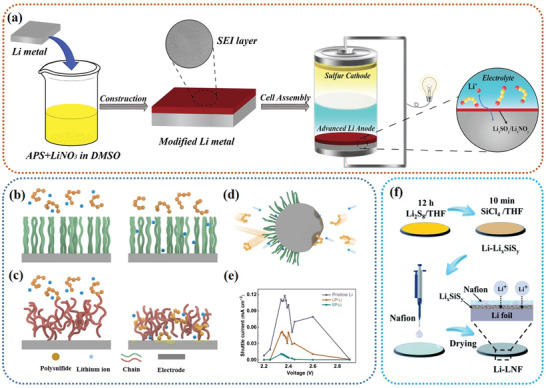
a) Schematic illustration of the fabrication for the modified Li metal and its application in Li–S batteries. Reproduced with permission.^[^ ^198^ ^]^ Copyright 2018, American Chemical Society. b–e) Schematic illustration of the diffusion behavior of Li^+^ ions through polymer interphases. The polysulfides are rejected by b) the ordered selectively permeable polymer interphase but accepted by c) the disordered polymer interphase in a working cell. d) Schematic illustration of corrosion behavior with/without selectively permeable interphase. e) The shuttle currents test in Li–S cell with LiNO_3_‐free ether electrolyte. Reproduced with permission.^[^ ^192^ ^]^ Copyright 2021, Wiley‐VCH. f) The fabrication of a Li‐LNF electrode. Reproduced with permission.^[^ ^194^ ^]^ Copyright 2020, Royal Society of Chemistry.

Ultrathin Al_2_O_3_ film with a thickness of 14 nm was coated on the surface of Li anode by Naked's teams via atomic layer deposition for inhibiting the shuttle behavior of LiPSs. This thin film could help to diffuse Li^+^ ion to anode because of its high ion conductivity and short transport distance. Meanwhile, the chemical stability was certified via a long time with no color change under exposure to air.^[^ ^196a^ ^]^ Besides, Al_2_O_3_ protective layer also can adsorb soluble LiPSs chemically via polar bonds to alleviate Li corrosion.^[^ ^196b^ ^]^ LiF or its compounds were synthesized by means of the chemical reaction of fluorine gas,^[^ ^197a^ ^]^ gaseous Freon,^[^ ^197b^ ^]^ or ammonium fluoride (NH_4_F) in tetrahydrofuran (THF) solvent^[^ ^197c^ ^]^ etc. With high ion conductivity of about 3 × 10^−9^ S cm^−1^ and good chemical stability with electrolyte and LiPSs, LiF could occlude access to sulfides and afford paths for Li^+^ ion transport. Even decorated on the surface of 3D structured Li metal with a large contact area to LiPSs, the passivation layer still could prevent Li from corroding.^[^ ^197b^ ^]^ Polycrystalline Li_3_N with an ultrahigh ionic conductivity of 10^−3^ to 10^−4^ S cm^−1^ was prepared via a direct reaction between the Li metal and N_2_ gas. Benefited from rapid diffusion kinetics, the Li_3_N passivation layer was capable of promoting the transformation from insulative Li_2_S/Li_2_S_2_ to soluble Li_2_S*_x_* on the surface during Li plating.^[^ ^201^ ^]^ Similarly, a compact inorganic composite layer mainly containing Li_2_O, LiF, and Li_3_N was constructed via pretreating Li metal with ion liquid to enhance diffusion kinetics.^[^ ^195^ ^]^ Besides, the inorganic components (Li*_x_* SO*_y_* and Li*_x_* NO*_y_*,) commonly found in the SEI layer of anode surface when contacting with traditional LiNO_3_‐containing electrolyte in Li–S batteries can be formed by pretreating Li with oxidants of ammonium persulfate and lithium nitrate to avoid other complex phases formation, as shown in **Figure** **13**a.^[^ ^198^ ^]^ Expect for Li compounds, Li‐containing alloys also were introduced to establish a conductive artificial layer, e.g., Li–Al alloy coating,^[^ ^202^ ^]^ Li_2_Se/Li_2_S.^[^ ^203^ ^]^ Yu and co‐workers introduced Li_2_Se with high chemical properties and good Li^+^ ion migration ability (>10^−5^ S cm^−1^) to the Li anode surface, which could alleviate the polysulfide reduction by metallic Li and improve the electrochemical performance of Li–S batteries.

#### Organic Physical Protective Layer

A dense passivation layer composed of inorganic components with poor mechanical flexibility and ductility is vulnerable to stress caused by repeated volume changes of the electrode during cycling. Li tends to nucleate preferentially in the crack or defect sites where the current is aggregated, eventually leading to the rapid growth of Li dendrites. LiPSs will permeate via the cracks or defect sites to oxidize Li anode and form insoluble Li_2_S/Li_2_S_2_. Organic components usually bring high mechanical toughness and benefit to build a more stable protective layer on the Li metal surface. For instance, PEDOT‐co‐PEG conductive polymer layer with good mechanical properties strongly adhered on Li surface to obstacle the corrosion reaction, which would accelerate reversible transform from insulative Li_2_S/Li_2_S_2_ to soluble Li_2_S*_x_* and suppress the growth of Li dendrites.^[^ ^204^ ^]^ Poly (dimethylsiloxane) (PDMS) film was formed by immersing Li metal in PDMS‐contained DOL/DME solution. LiPSs and reactive species were separated physically and hard to get access to the Li surface.^[^ ^199^ ^]^ As depicted in Figure 13b–e, PDMS terminated with lithiophilic end groups of aminopropyl was utilized to construct selectively permeable ordered channels on Li surface, wherein Li^+^ ions could commute by means of the electrochemical overpotential and then LiPSs shuttle was blocked owing to the much larger gyration volume than Li^+^ ions. Li–S pouch cells coupling with a selectively permeable layer could achieve a low shuttle current and a prolonged cycle life of 75 cycles with high Coulombic efficiency of 99%.^[^ ^192^ ^]^ Another Li^+^ permeable layer was formed by pretreating Li metal with benzo‐substituted crown ether in an electrolyte, i.e., benzo‐15‐crown‐5. By benzo group substitution, the electron‐donor ability of ring oxygens was weakened to improve Li^+^ mobility, and S*_x_* ^2–^ anions are blocked to prevent contact with Li anode.^[^ ^205^ ^]^

#### Protective Layer with Interaction Force

Unlike physical barriers, repelling LiPSs through interaction force can avoid the accumulation of insoluble Li_2_S*_x_*. Nevertheless, to avoid the repulsion to Li^+^ ions, the interaction should be selective, allowing the hopping of cations but prevents intrusion of anions.^[^ ^147^ ^]^ E.g., cation permselective layer consisting of Li terminated sulfonated TiO_2_ (LTST) nanoparticles can shield the permeation of polysulfide anions via electrostatic interaction.^[^ ^206^ ^]^ The robust conformal coating as an electrostatic shield maintains high conductivity by tethering Li and prevents interfacial aggregation of soluble anionic species at the anode by negatively charged/polar sulfonate groups. In addition, Nafion with highly cation‐selective properties has been introduced to repel LiPSs.^[^ ^194^, ^200^, ^207^ ^]^ A dual‐layered artificial SEI on the surface of Li anode consists of organic lithiated Nafion on the top and inorganic Li*_x_* SiS*_y_* (including Li_2_SiS_3_/Li_4_SiS_4_, SiS_2,_ and Li_2_S) on the bottom, as shown in Figure 13f. The organic Nafion layer with good flexibility and sulfonate groups (—SO_3_^–^) can maintain the stability of the SEI layer and suppress Li dendrite growth, as well as block polysulfides to shuttle. While the rigid inorganic layer can enhance Li^+^ ion conductivity and separate Li anode from the electrolyte.^[^ ^194^ ^]^ However, Nafions would suffer from swell and dissolution problems. The introduction of PVDF helps to entangle and entrap Nafions molecules within the Nafion/PVDF composite layer. And the polarized C—F bonds in PVDF and sulfonic functional groups in Nafions can repel polysulfides away from Li anode synergistically, suppressing self‐discharge behavior.^[^ ^200^ ^]^

### 3.5 Summary

Based on the above discussions, it is clear that each component in cells has its own magical way of dealing with the notorious shuttle effect, and strategies for suppressing the shuttle phenomenon can be divided into four parts in Li–S batteries. The sulfur cathode plays a vital role as the active sites of active sulfur storage and reaction, and the root cause of the polysulfide shuttle problems in fact lies within the sulfur cathode. Rationally designed cathodes can block the dissolution and diffusion behavior of polysulfides from the origin. But the understanding of the electrocatalytic mechanism is still ambiguous currently, as well as high sulfur content, high areal sulfur loading, and lightweight requirements still cloud the development of cathodes. As for electrolytes, although liquid electrolytes, gel electrolytes, and some solid‐state electrolytes cannot get rid of the shuttle effect completely, the utilization of ideal all‐solid‐state electrolytes with sulfide‐solid electrolytes or composite solid electrolytes without polymers/liquid components can achieve the one‐step solid‐solid conversion reaction during discharge, thus realizing the actually shuttle‐free Li–S batteries. However, there are still many problems in solid‐state electrolytes that need to be solved urgently, such as low ionic conductivity, high interfacial resistance, and narrow voltage window. Only such challenges are settled well, the solid‐state electrolytes with excellent and stable properties would be applied in Li–S batteries.

The separator coating layers, as a complement and reinforcement of the sulfur hosts, can also be regarded as a secondary collector. The introduction of such a second barrier modification layer can directly prevent polysulfides from penetrating across the separator. Besides, the polysulfides adsorbed on the modified layer can be reactivated electrochemically to participate in the subsequent electrochemical reactions, thus enhancing the utilization of sulfur. To achieve such effects, the volume loading, mass loading, thickness, and wetting properties towards electrolytes, etc. should be well considered. Finally, as the final hurdle in the polysulfides shuttle process, the anode surface engineering is more like a final selectable shielded door, ensuring smooth Li^+^ ions transport while isolating polysulfides from direct contact with Li metal, thus suppressing the Li metal corrosion by soluble LiPSs and reducing the unnecessary sulfur loss. Meanwhile, the concerns about the instability of the SEI layer that would crack into fragments easily during cycling should be also taken into consideration.

Overall, when breaking down the components of a Li–S battery, each component has its own unique advantages to combat the shuttle effect, and the improvements in each component can achieve corresponding performance enhancement. There is still no optimal solution to the shuttle effect, but it is clear that a collaborative effort is needed to boost the performance of the batteries.

## 4 Conclusion and Future Outlooks

Li–S batteries are considered to be the most promising next‐generation energy storage systems owing to their ultrahigh energy density and low cost of the sulfur raw materials. However, the commercialization of Li–S batteries is still hindered by the low Coulombic efficiency, rapid capacity decay, and potential safety risks caused by the shuttle effect. As long as the shuttle behaviors of LiPSs exist during the operation of Li–S batteries, the battery's performance is too difficult to satisfy the practical demand. Therefore, a deeper and more comprehensive understanding of the LiPSs shuttle process is an urgent need and meaningful, which can also provide references for the future design of the shuttle effect inhibition toward the high‐performance Li–S batteries.

In this review, we concentrate on the shuttle path of LiPSs in a Li–S battery and their influences on the corresponding components, retrospecting the recent advances and strategies of the sulfur host, electrolyte system, separator, and anode protection toward the shuttle effect inhibition. Such strategies include: 1) Immobilizing and/or electrocatalyzing the LiPSs conversion by modified sulfur hosts. From physical adsorption to chemical adsorption, and further chemically catalyzing the conversion of LiPSs, the anchoring effect of sulfur cathodes on polysulfides has been continuously strengthened and favorable results have been achieved. 2) Reducing solubility or eliminating the formation of LiPSs by tailored electrolyte systems. The composition tailoring of the liquid electrolyte can effectively prevent the dissolution of polysulfides into the electrolyte, and the use of solid‐state electrolyte to assemble all‐solid‐state batteries can avoid the formation of LiPSs and thus eliminate the shuttle effect. 3) Blocking the LiPSs from permeating the separators by modified layers. The modification layer on the separator can work as a physical barrier to effectively grasp the LiPSs that reach the separator surface and restrict them on the cathode side. Electrochemical catalysis can promote their conversion and keep them participating in subsequent electrochemical reactions. 4) Preventing the surface chemical reaction between LiPSs and Li anode by anode interface engineerings. Through the design of the protective layer, including the in‐suit SEI layers and artificial protective layers, the direct contact and interface chemical reactions between LiPSs and Li metal can be effectively prevented, thereby avoiding unnecessary sulfur loss and passivation of the Li metal surface.

Although various strategies proposed to inhibit the shuttle effect of polysulfides have achieved corresponding progress and showed favorable effects in improving the electrochemical performance of Li–S batteries, there are still many problems and difficulties that should be considered to realize the large‐scale commercial application in the near future.

1) In depth exploration of catalytic mechanism: Although the catalysis of mediators in Li–S chemistry has been deeply rooted in the hearts of the people, and a large amount of experimental data and phenomena have proved such catalysis effects, the specific mechanism has not yet been elucidated due to the multistage and complex reaction process. There is still a lack of deep understanding of the ion/electron transfer and conversion at the working surface/interface of the electrocatalyst. And the spatial distribution and nucleation behavior of the Li_2_S discharge product and S_8_ oxidation product is still ambiguous. Only a deeper understanding of the adsorption‐catalytic mechanism at the molecular or atomic level can provide a more direct presentation about the catalysis in Li–S batteries. Fortunately, with the continuous development of characterization techniques, the mystery of the catalysis mechanism is gradually being unveiled.^[^ ^208^ ^]^ In situ characterization techniques such as operando XAS, IR, XPS, and STEM should be developed and optimized to real‐time monitor the instantaneous change and evolution process inside the battery during the operation process, thereby providing accurate guidance for the design of advanced mediators materials for Li–S batteries.
2) Development of polysulfides‐free solid‐state electrolyte: Replacing liquid electrolytes with all solid‐state electrolytes is the most effective strategy to overcome the polysulfides shuttle problem intrinsically. Of which, oxide‐based and solid polymer electrolytes are usually combined with liquid solvent or polymer, resulting in an unavoidable polysulfides formation. Sulfide‐solid electrolytes or composite solid electrolytes are considered to be the best choice for the elimination of polysulfides shuttle due to the absence of liquid components. However, low ionic conductivity, high interfacial resistance, chemical, and electrochemical instability are insurmountable troubles faced by all‐solid‐state Li–S batteries. Only with these tough problems are better solved can the application of all‐solid‐state Li–S batteries become possible and solve the shuttle effect fundamentally.
3) Exploration on full cell and practical pouch cell: In coin‐type half cell research, the thick Li plates are used as anodes, in which, the negative effects of LiPSs on Li anode are usually neglected under a high N/P ratio (>150).^[^ ^162^ ^]^ However, as for Li–S full cells, especially for practical pouch cells, the fatal deterioration of Li metal anode and lack of Li supply are crucial reasons for the dramatic capacity loss and rapid battery failure.^[^ ^163^ ^]^ The limited Li mass loading in the pouch cell with a low N/P ratio cannot afford continuous irreversible Li consumption caused by reacting with LiPSs. And high LiPSs concentration induced by high sulfur loading causes more severe shuttle effect and Li corrosion. Thus, while making every effort to solve the shuttle effect problem, the protection of lithium metal and the inhibition of dendrite growth, etc., should also be considered in full cells. In addition, to pave the way for practical applications of Li–S batteries, attention needs to be paid not only to materials research but also to electrode architecture and cell engineering.
4) Matching for the large‐scale practical application: High sulfur content, high areal mass loading, as well as low electrolyte/sulfur (E/S) ratio, have profound impacts on the energy density of Li–S batteries. Indeed, an estimated 7 mg cm^−2^ of sulfur loading is the minimum requirement to achieve 300 Wh L^−1^.^[^ ^209^ ^]^ Nevertheless, the greater sulfur content is accompanied by more LiPSs production, and the small amount of electrolyte also poses greater challenges to wettability and ion migration, which puts higher requirements on cell components. In addition to the internal conditions, such parameters as the pressure and temperature range used, storage conditions, etc. are all key factors that need to be further explored during the practical application. Importantly, as a potential safety hazard, the thermal stability of Li–S batteries has been seldom studied and should be taken seriously and explored systematically. Moreover, although sulfur is not toxic, the recovery of Li metal resources of Li–S batteries after the large‐scale commercial application is still a tricky challenge.

In the end, although considerable progress has been made in the development of Li–S batteries, there are still many challenges to be solved in the process of commercialization. It is believed that with the upgrading of characterization technologies and the in‐depth exploration of reaction mechanisms, we can comprehensively and thoroughly understand lithium–sulfur batteries from a more microscopic point of view, and further effectively eliminate the shuttle effect.

## Conflict of Interest

The authors declare no conflict of interest.

## References

[1] a) M. Armand , J.‐M. Tarascon , Nature 2008, 451, 652; 18256660 10.1038/451652a

[2] a) D. Lin , Y. Liu , Y. Cui , Nat. Nanotechnol. 2017, 12, 194; 28265117 10.1038/nnano.2017.16

[3] H.‐J. Peng , J.‐Q. Huang , X.‐B. Cheng , Q. Zhang , Adv. Energy Mater. 2017, 7, 1700260.

[4] a) X. Ji , K. T. Lee , L. F. Nazar , Nat. Mater. 2009, 8, 500; 19448613 10.1038/nmat2460

[5] W. Deng , J. Phung , G. Li , X. Wang , Nano Energy 2021, 82, 105761.

[6] a) W. Ren , W. Ma , S. Zhang , B. Tang , Energy Storage Mater. 2019, 23, 707;

[7] J. Y. J. L. Wang , J. Y. Xie , N. X. Xu , Y. Li , Electrochem. Commun. 2002, 4, 499.

[8] R. Y. Yan , M. Oschatz , F. X. Wu , Carbon 2020, 161, 162.

[9] F. Wu , F. Chu , G. A. Ferrero , M. Sevilla , A. B. Fuertes , O. Borodin , Y. Yu , G. Yushin , Nano Lett. 2020, 20, 5391. 32463248 10.1021/acs.nanolett.0c01778

[10] S. Dörfler , H. Althues , P. Härtel , T. Abendroth , B. Schumm , S. Kaskel , Joule 2020, 4, 539.

[11] J.‐J. Chen , R.‐M. Yuan , J.‐M. Feng , Q. Zhang , J.‐X. Huang , G. Fu , M.‐S. Zheng , B. Ren , Q.‐F. Dong , Chem. Mater. 2015, 27, 2048.

[12] Y. Yang , G. Zheng , S. Misra , J. Nelson , M. F. Toney , Y. Cui , J. Am. Chem. Soc. 2012, 134, 15387. 22909273 10.1021/ja3052206

[13] a) L. Zhou , D. L. Danilov , R.‐A. Eichel , P. H. L. Notten , Adv. Energy Mater. 2021, 11, 2001304;

[14] G. Babu , K. Ababtain , K. Y. Ng , L. M. Arava , Sci. Rep. 2015, 5, 8763. 25740731 10.1038/srep08763 PMC4350110

[15] S. Xin , L. Gu , N. H. Zhao , Y. X. Yin , L. J. Zhou , Y. G. Guo , L. J. Wan , J. Am. Chem. Soc. 2012, 134, 18510. 23101502 10.1021/ja308170k

[16] Z. Li , L. Yuan , Z. Yi , Y. Sun , Y. Liu , Y. Jiang , Y. Shen , Y. Xin , Z. Zhang , Y. Huang , Adv. Energy Mater. 2014, 4, 1301473.

[17] Z. Li , Z. Xiao , S. Wang , Z. Cheng , P. Li , R. Wang , Adv. Funct. Mater. 2019, 29, 1902322.

[18] G. Zheng , Y. Yang , J. J. Cha , S. S. Hong , Y. Cui , Nano Lett. 2011, 11, 4462. 21916442 10.1021/nl2027684

[19] J. H. Yun , J.‐H. Kim , D. K. Kim , H.‐W. Lee , Nano Lett. 2018, 18, 475. 29235876 10.1021/acs.nanolett.7b04425

[20] Y. Li , X.‐T. Guo , S.‐T. Zhang , H. Pang , Rare Met. 2020, 40, 417.

[21] H. Chen , G. Zhou , D. Boyle , J. Wan , H. Wang , D. Lin , D. Mackanic , Z. Zhang , S. C. Kim , H. R. Lee , H. Wang , W. Huang , Y. Ye , Y. Cui , Matter 2020, 2, 1605.

[22] H. Li , X. Yang , X. Wang , M. Liu , F. Ye , J. Wang , Y. Qiu , W. Li , Y. Zhang , Nano Energy 2015, 12, 468.

[23] a) G. Zhou , L. C. Yin , D. W. Wang , L. Li , S. Pei , I. R. Gentle , F. Li , H. M. Cheng , ACS Nano 2013, 7, 5367; 23672616 10.1021/nn401228t

[24] J. Wang , X. Yan , Z. Zhang , H. Ying , R. Guo , W. Yang , W.‐Q. Han , Adv. Funct. Mater. 2019, 29, 2006798.

[25] Z. Shi , M. Li , J. Sun , Z. Chen , Adv. Energy Mater. 2021, 11, 2100332.

[26] X. J. Zhou , J. Tian , Q. P. Wu , J. L. Hu , C. L. Li , Energy Storage Mater. 2020, 24, 644.

[27] J. Wang , H. Yang , Z. Chen , L. Zhang , J. Liu , P. Liang , H. Yang , X. Shen , Z. X. Shen , Adv. Sci. 2018, 5, 1800621. 10.1002/advs.201800621 PMC6247042 30479918

[28] Z. Liang , G. Zheng , W. Li , Z. W. Seh , H. Yao , K. Yan , D. Kong , Y. Cui , ACS Nano 2014, 8, 5249. 24766547 10.1021/nn501308m

[29] a) W. W. Yang , Y. Wei , Q. Chen , S. J. Qin , J. H. Zuo , S. D. Tan , P. B. Zhai , S. Q. Cui , H. W. Wang , C. Q. Jin , J. Xiao , W. Liu , J. X. Shang , Y. J. Gong , J. Mater. Chem. A 2020, 8, 15816;

[30] T. Pan , Z. Li , Q. He , X. Xu , L. He , J. Meng , C. Zhou , Y. Zhao , L. Mai , Energy Storage Mater. 2019, 23, 55.

[31] Z. Xiao , Z. Li , P. Li , X. Meng , R. Wang , ACS Nano 2019, 13, 3608. 30864777 10.1021/acsnano.9b00177

[32] L. Guan , H. Hu , L. Li , Y. Pan , Y. Zhu , Q. Li , H. Guo , K. Wang , Y. Huang , M. Zhang , Y. Yan , Z. Li , X. Teng , J. Yang , J. Xiao , Y. Zhang , X. Wang , M. Wu , ACS Nano 2020, 14, 6222. 32352746 10.1021/acsnano.0c02294

[33] S. Liu , T. Zhao , X. Tan , L. Guo , J. Wu , X. Kang , H. Wang , L. Sun , W. Chu , Nano Energy 2019, 63, 103894.

[34] L. Lin , F. Pei , J. Peng , A. Fu , J. Cui , X. Fang , N. Zheng , Nano Energy 2018, 54, 50.

[35] H. Chen , C. Wang , Y. Dai , S. Qiu , J. Yang , W. Lu , L. Chen , Nano Lett. 2015, 15, 5443. 26148126 10.1021/acs.nanolett.5b01837

[36] K. Xie , Y. You , K. Yuan , W. Lu , K. Zhang , F. Xu , M. Ye , S. Ke , C. Shen , X. Zeng , X. Fan , B. Wei , Adv. Mater. 2017, 29, 1604724. 10.1002/adma.201604724 27918119

[37] F. Wu , H. Lv , S. Chen , S. Lorger , V. Srot , M. Oschatz , P. A. van Aken , X. Wu , J. Maier , Y. Yu , Adv. Funct. Mater. 2019, 29, 1902820.

[38] Y. Hao , L. Wang , Y. Liang , B. He , Y. Zhang , B. Cheng , W. Kang , N. Deng , Nanoscale 2019, 11, 21324. 31670739 10.1039/c9nr07809f

[39] M. Li , K. Fu , Z. Wang , C. Cao , J. Yang , Q. Zhai , Z. Zhou , J. Ji , Y. Xue , C. Tang , Chem. ‐ Eur. J. 2020, 26, 17567. 32965742 10.1002/chem.202003807

[40] Z. L. Xu , S. Lin , N. Onofrio , L. Zhou , F. Shi , W. Lu , K. Kang , Q. Zhang , S. P. Lau , Nat. Commun. 2018, 9, 4164. 30301957 10.1038/s41467-018-06629-9 PMC6177446

[41] M. Yu , J. Ma , M. Xie , H. Song , F. Tian , S. Xu , Y. Zhou , B. Li , D. Wu , H. Qiu , R. Wang , Adv. Energy Mater. 2017, 7, 1602347.

[42] Q. Pang , D. Kundu , M. Cuisinier , L. F. Nazar , Nat. Commun. 2014, 5, 4759. 25154399 10.1038/ncomms5759

[43] J. Zhang , Z. Li , Y. Chen , S. Gao , X. W. Lou , Angew. Chem., Int. Ed. 2018, 57, 10944. 10.1002/anie.201805972 29949224

[44] X. Wang , C. Yang , X. Xiong , G. Chen , M. Huang , J.‐H. Wang , Y. Liu , M. Liu , K. Huang , Energy Storage Mater. 2019, 16, 344.

[45] Q. Zhang , Z. Qiao , X. Cao , B. Qu , J. Yuan , T. E. Fan , H. Zheng , J. Cui , S. Wu , Q. Xie , D.‐L. Peng , Nanoscale Horiz. 2020, 5, 720. 32053127 10.1039/c9nh00663j

[46] R. Wang , J. Yang , X. Chen , Y. Zhao , W. Zhao , G. Qian , S. Li , Y. Xiao , H. Chen , Y. Ye , G. Zhou , F. Pan , Adv. Energy Mater. 2020, 10, 1903550.

[47] Z. Qiao , F. Zhou , Q. Zhang , F. Pei , H. Zheng , W. Xu , P. Liu , Y. Ma , Q. Xie , L. Wang , X. Fang , D.‐L. Peng , Energy Storage Mater. 2019, 23, 62.

[48] Z. Shi , Z. Sun , J. Cai , Z. Fan , J. Jin , M. Wang , J. Sun , Adv. Funct. Mater. 2020, 31, 2006798.

[49] Z. Yang , C. Peng , R. Meng , L. Zu , Y. Feng , B. Chen , Y. Mi , C. Zhang , J. Yang , ACS Cent. Sci. 2019, 5, 1876. 31807689 10.1021/acscentsci.9b00846 PMC6891857

[50] Z. Zhang , D. Luo , G. Li , R. Gao , M. Li , S. Li , L. Zhao , H. Dou , G. Wen , S. Sy , Y. Hu , J. Li , A. Yu , Z. Chen , Matter 2020, 3, 920.

[51] Y. Wang , R. Zhang , J. Chen , H. Wu , S. Lu , K. Wang , H. Li , C. J. Harris , K. Xi , R. V. Kumar , S. Ding , Adv. Energy Mater. 2019, 9, 1900953.

[52] H. Chen , W.‐D. Dong , F.‐J. Xia , Y.‐J. Zhang , M. Yan , J.‐P. Song , W. Zou , Y. Liu , Z.‐Y. Hu , J. Liu , Y. Li , H.‐E. Wang , L.‐H. Chen , B.‐L. Su , Chem. Eng. J. 2020, 381, 122746.

[53] Z. Yuan , H.‐J. Peng , T.‐Z. Hou , J.‐Q. Huang , C.‐M. Chen , D.‐W. Wang , X.‐B. Cheng , F. Wei , Q. Zhang , Nano Lett. 2016, 16, 519. 26713782 10.1021/acs.nanolett.5b04166

[54] G. Liu , D. Luo , R. Gao , Y. Hu , A. Yu , Z. Chen , Small 2020, 16, 2001089. 10.1002/smll.202001089 32776459

[55] H. Wang , Q. Zhang , H. Yao , Z. Liang , H. W. Lee , P. C. Hsu , G. Zheng , Y. Cui , Nano Lett. 2014, 14, 7138. 25372985 10.1021/nl503730c

[56] H. Lin , L. Yang , X. Jiang , G. Li , T. Zhang , Q. Yao , G. W. Zheng , J. Y. Lee , Energy Environ. Sci. 2017, 10, 1476.

[57] L. Luo , J. Li , H. Yaghoobnejad Asl , A. Manthiram , ACS Energy Lett. 2020, 5, 1177.

[58] D. Yang , C. Zhang , J. J. Biendicho , X. Han , Z. Liang , R. Du , M. Li , J. Li , J. Arbiol , J. Llorca , Y. Zhou , J. R. Morante , A. Cabot , ACS Nano 2020, 14, 15492. 33084302 10.1021/acsnano.0c06112

[59] Z. Ye , Y. Jiang , L. Li , F. Wu , R. Chen , Adv. Mater. 2020, 32, 2002168. 10.1002/adma.202002168 32596845

[60] G. Chen , Y. Li , W. Zhong , F. Zheng , J. Hu , X. Ji , W. Liu , C. Yang , Z. Lin , M. Liu , Energy Storage Mater. 2020, 25, 547.

[61] Y. Zhang , G. Li , J. Wang , G. Cui , X. Wei , L. Shui , K. Kempa , G. Zhou , X. Wang , Z. Chen , Adv. Funct. Mater. 2020, 30, 2001165.

[62] S. Shen , X. Xia , Y. Zhong , S. Deng , D. Xie , B. Liu , Y. Zhang , G. Pan , X. Wang , J. Tu , Adv. Mater. 2019, 31, 1900009. 10.1002/adma.201900009 30843629

[63] Z. Sun , J. Zhang , L. Yin , G. Hu , R. Fang , H.‐M. Cheng , F. Li , Nat. Commun. 2017, 8, 14627. 28256504 10.1038/ncomms14627 PMC5337987

[64] N. Li , Z. Xu , P. Wang , Z. Zhang , B. Hong , J. Li , Y. Lai , Chem. Eng. J. 2020, 398, 122746.

[65] Y. Wang , R. Zhang , Y.‐c. Pang , X. Chen , J. Lang , J. Xu , C. Xiao , H. Li , K. Xi , S. Ding , Energy Storage Mater. 2019, 16, 228.

[66] W. Sun , C. Liu , Y. Li , S. Luo , S. Liu , X. Hong , K. Xie , Y. Liu , X. Tan , C. Zheng , ACS Nano 2019, 13, 12137. 31593436 10.1021/acsnano.9b06629

[67] Z. Sun , S. Vijay , H. H. Heenen , A. Y. S. Eng , W. Tu , Y. Zhao , S. W. Koh , P. Gao , Z. W. Seh , K. Chan , H. Li , Adv. Energy Mater. 2020, 10, 1904010.

[68] D.‐R. Deng , F. Xue , Y.‐J. Jia , J.‐C. Ye , C.‐D. Bai , M.‐S. Zheng , Q.‐F. Dong , ACS Nano 2017, 11, 6031. 28570815 10.1021/acsnano.7b01945

[69] Z. Qiao , Y. Zhang , Z. Meng , Q. Xie , L. Lin , H. Zheng , B. Sa , J. Lin , L. Wang , D.‐L. Peng , Adv. Funct. Mater. 2021, 31, 2100970.

[70] T. Zhou , W. Lv , J. Li , G. Zhou , Y. Zhao , S. Fan , B. Liu , B. Li , F. Kang , Q.‐H. Yang , Energy Environ. Sci. 2017, 10, 1694.

[71] R. Wang , C. Luo , T. Wang , G. Zhou , Y. Deng , Y. He , Q. Zhang , F. Kang , W. Lv , Q.‐H. Yang , Adv. Mater. 2020, 32, 2000315. 10.1002/adma.202000315 32627911

[72] P. Zhu , J. Zhu , C. Yan , M. Dirican , J. Zang , H. Jia , Y. Li , Y. Kiyak , H. Tan , X. Zhang , Adv. Mater. Interfaces 2018, 5, 1701598.

[73] J. Yan , B. Li , X. Liu , Nano Energy 2015, 18, 245.

[74] a) W. G. Lim , S. Kim , C. Jo , J. Lee , Angew. Chem., Int. Ed. 2019, 58, 18746; 10.1002/anie.201902413 31069949

[75] C. Deng , Z. Wang , L. Feng , S. Wang , J. Yu , J. Mater. Chem. A 2020, 8, 19704.

[76] Y. Song , W. Cai , L. Kong , J. Cai , Q. Zhang , J. Sun , Adv. Energy Mater. 2019, 10, 1901075.

[77] C. Li , J. B. Baek , ACS Omega 2020, 5, 31. 32803082 10.1021/acsomega.0c02838 PMC7424710

[78] Z.‐W. Zhang , H.‐J. Peng , M. Zhao , J.‐Q. Huang , Adv. Funct. Mater. 2018, 28, 1707536.

[79] H. Al Salem , G. Babu , C. V. Rao , L. M. Arava , J. Am. Chem. Soc. 2015, 137, 11542. 26331670 10.1021/jacs.5b04472

[80] H. Li , L. Fei , R. Zhang , S. Yu , Y. Zhang , L. Shu , Y. Li , Y. Wang , J. Energy Chem. 2020, 49, 339.

[81] J. He , A. Bhargav , A. Manthiram , ACS Nano 2021, 15, 8583. 33891408 10.1021/acsnano.1c00446

[82] G. Zhou , S. Zhao , T. Wang , S.‐Z. Yang , B. Johannessen , H. Chen , C. Liu , Y. Ye , Y. Wu , Y. Peng , C. Liu , S. P. Jiang , Q. Zhang , Y. Cui , Nano Lett. 2020, 20, 1252. 31887051 10.1021/acs.nanolett.9b04719

[83] Y. Li , G. Chen , J. Mou , Y. Liu , S. Xue , T. Tan , W. Zhong , Q. Deng , T. Li , J. Hu , C. Yang , K. Huang , M. Liu , Energy Storage Mater. 2020, 28, 196.

[84] H. Shi , X. Ren , J. Lu , C. Dong , J. Liu , Q. Yang , J. Chen , Z. S. Wu , Adv. Energy Mater. 2020, 10, 2002271.

[85] Y. Wang , D. Adekoya , J. Sun , T. Tang , H. Qiu , L. Xu , S. Zhang , Y. Hou , Adv. Funct. Mater. 2018, 29, 1807485.

[86] X. Liu , J.‐Q. Huang , Q. Zhang , L. Mai , Adv. Mater. 2017, 29, 1601759. 10.1002/adma.201601759 28160327

[87] X. Tao , J. Wang , C. Liu , H. Wang , H. Yao , G. Zheng , Z. W. Seh , Q. Cai , W. Li , G. Zhou , C. Zu , Y. Cui , Nat. Commun. 2016, 7, 11203. 27046216 10.1038/ncomms11203 PMC4822044

[88] S. Xin , J. Li , H. Cui , Y. Liu , H. Wei , Y. Zhong , M. Wang , Chem. Eng. J. 2021, 410, 128153.

[89] H. Chen , W.‐D. Dong , F.‐J. Xia , Y.‐J. Zhang , M. Yan , J.‐P. Song , W. Zou , Y. Liu , Z.‐Y. Hu , J. Liu , Y. Li , H.‐E. Wang , L.‐H. Chen , B.‐L. Su , Chem. Eng. J. 2020, 381, 122746.

[90] M. Yu , W. Yuan , C. Li , J.‐D. Hong , G. Shi , J. Mater. Chem. A 2014, 2, 7360.

[91] T. Liu , Y. Zhang , C. H. Li , M. D. Marquez , H. V. Tran , F. C. Robles Hernandez , Y. Yao , T. R. Lee , ACS Appl. Mater. Interfaces 2020, 12, 47368. 32930564 10.1021/acsami.0c10341

[92] X. Liang , C. Y. Kwok , F. Lodi‐Marzano , Q. Pang , M. Cuisinier , H. Huang , C. J. Hart , D. Houtarde , K. Kaup , H. Sommer , T. Brezesinski , J. Janek , L. F. Nazar , Adv. Energy Mater. 2016, 6, 2003689.

[93] K. Xi , D. He , C. Harris , Y. Wang , C. Lai , H. Li , P. R. Coxon , S. Ding , C. Wang , R. V. Kumar , Adv. Sci. 2019, 6, 1800815. 10.1002/advs.201800815 PMC6425436 30937253

[94] Y. Boyjoo , H. Shi , E. Olsson , Q. Cai , Z. S. Wu , J. Liu , G. Q. Lu , Adv. Energy Mater. 2020, 10, 2000651.

[95] Y. Wu , X. Zhu , P. Li , T. Zhang , M. Li , J. Deng , Y. Huang , P. Ding , S. Wang , R. Zhang , J. Lu , G. Lu , Y. Li , Y. Li , Nano Energy 2019, 59, 636.

[96] H.‐J. Peng , G. Zhang , X. Chen , Z.‐W. Zhang , W.‐T. Xu , J.‐Q. Huang , Q. Zhang , Angew. Chem., Int. Ed. 2016, 55, 12990. 10.1002/anie.201605676 27513988

[97] R. T. Wang , J. W. Lang , P. Zhang , Z. Y. Lin , X. B. Yan , Adv. Funct. Mater. 2015, 25, 2270.

[98] Y. Zhong , D. Chao , S. Deng , J. Zhan , R. Fang , Y. Xia , Y. Wang , X. Wang , X. Xia , J. Tu , Adv. Funct. Mater. 2018, 28, 1706391.

[99] a) H. Ye , J. Sun , S. Zhang , H. Lin , T. Zhang , Q. Yao , J. Y. Lee , ACS Nano 2019, 13, 14208; 31790591 10.1021/acsnano.9b07121

[100] N. Wang , B. Chen , K. Qin , E. Liu , C. Shi , C. He , N. Zhao , Nano Energy 2019, 60, 332.

[101] B. Li , Q. Su , L. Yu , J. Zhang , G. Du , D. Wang , D. Han , M. Zhang , S. Ding , B. Xu , ACS Nano 2020, 14, 17285. 10.1021/acsnano.0c07332 33211956

[102] J. Zhou , X. Liu , L. Zhu , J. Zhou , Y. Guan , L. Chen , S. Niu , J. Cai , D. Sun , Y. Zhu , J. Du , G. Wang , Y. Qian , Joule 2018, 2, 2681.

[103] Q. Pang , C. Y. Kwok , D. Kundu , X. Liang , L. F. Nazar , Joule 2019, 3, 136.

[104] D. Luo , Z. Zhang , G. Li , S. Cheng , S. Li , J. Li , R. Gao , M. Li , S. Sy , Y. P. Deng , Y. Jiang , Y. Zhu , H. Dou , Y. Hu , A. Yu , Z. Chen , ACS Nano 2020, 14, 4849. 32182038 10.1021/acsnano.0c00799

[105] W. Tian , B. Xi , Z. Feng , H. Li , J. Feng , S. Xiong , Adv. Energy Mater. 2019, 9, 1901896.

[106] Y. Liu , A. K. Haridas , K.‐K. Cho , Y. Lee , J.‐H. Ahn , J. Phys. Chem. C 2017, 121, 26172.

[107] Y. Yang , G. Yu , J. J. Cha , H. Wu , M. Vosgueritchian , Y. Yao , Z. Bao , Y. Cui , ACS Nano 2011, 5, 9187. 21995642 10.1021/nn203436j

[108] W. Li , Q. Zhang , G. Zheng , Z. W. Seh , H. Yao , Y. Cui , Nano Lett. 2013, 13, 5534. 24127640 10.1021/nl403130h

[109] J. Wang , J. Yang , J. Xie , N. Xu , Adv. Mater. 2002, 14, 963.

[110] G. Zhou , J. Sun , Y. Jin , W. Chen , C. Zu , R. Zhang , Y. Qiu , J. Zhao , D. Zhuo , Y. Liu , X. Tao , W. Liu , K. Yan , H. R. Lee , Y. Cui , Adv. Mater. 2017, 29, 1603366. 10.1002/adma.201603366 28134456

[111] T. Yim , M.‐S. Park , J.‐S. Yu , K. J. Kim , K. Y. Im , J.‐H. Kim , G. Jeong , Y. N. Jo , S.‐G. Woo , K. S. Kang , I. Lee , Y.‐J. Kim , Electrochim. Acta 2013, 107, 454.

[112] M. Cuisinier , P. E. Cabelguen , B. D. Adams , A. Garsuch , M. Balasubramanian , L. F. Nazar , Energy Environ. Sci. 2014, 7, 2697.

[113] S. Gu , R. Qian , J. Jin , Q. Wang , J. Guo , S. Zhang , S. Zhuo , Z. Wen , Phys. Chem. Chem. Phys. 2016, 18, 29293. 27731873 10.1039/c6cp04775k

[114] J. Zheng , G. Ji , X. Fan , J. Chen , Q. Li , H. Wang , Y. Yang , K. C. DeMella , S. R. Raghavan , C. Wang , Adv. Energy Mater. 2019, 9, 1803774.

[115] a) C. Zhao , G. L. Xu , T. Zhao , K. Amine , Angew. Chem., Int. Ed. 2020, 59, 17634; 10.1002/anie.202007159 32645250

[116] S. Chen , F. Dai , M. L. Gordin , Z. Yu , Y. Gao , J. Song , D. Wang , Angew. Chem., Int. Ed. 2016, 55, 4231. 10.1002/anie.201511830 26918660

[117] T. Yang , T. Qian , J. Liu , N. Xu , Y. Li , N. Grundish , C. Yan , J. B. Goodenough , ACS Nano 2019, 13, 9067. 31339690 10.1021/acsnano.9b03304

[118] L. Suo , Y. S. Hu , H. Li , M. Armand , L. Chen , Nat. Commun. 2013, 4, 1481. 23403582 10.1038/ncomms2513

[119] R. Amine , J. Liu , I. Acznik , T. Sheng , K. Lota , H. Sun , C. J. Sun , K. Fic , X. Zuo , Y. Ren , D. A. Ei‐Hady , W. Alshitari , A. S. Al‐Bogami , Z. Chen , K. Amine , G. L. Xu , Adv. Energy Mater. 2020, 10, 2000901.

[120] X. Yu , Z. Bi , F. Zhao , A. Manthiram , Adv. Energy Mater. 2016, 6, 1601392.

[121] Q. Wang , J. Jin , X. Wu , G. Ma , J. Yang , Z. Wen , Phys. Chem. Chem. Phys. 2014, 16, 21225. 25198434 10.1039/c4cp03694h

[122] L. Wang , Y. Wang , Y. Xia , Energy Environ. Sci. 2015, 8, 1551.

[123] Z. Fan , B. Ding , T. Zhang , Q. Lin , V. Malgras , J. Wang , H. Dou , X. Zhang , Y. Yamauchi , Small 2019, 15, 1903952. 10.1002/smll.201903952 31565864

[124] L. Chen , L. Z. Fan , Energy Storage Mater. 2018, 15, 37.

[125] C. Zhang , Y. Lin , J. Liu , J. Mater. Chem. A 2015, 3, 10760.

[126] Y. Lin , X. Wang , J. Liu , J. D. Miller , Nano Energy 2017, 31, 478.

[127] J. Liang , Q. Sun , Y. Zhao , Y. Sun , C. Wang , W. Li , M. Li , D. Wang , X. Li , Y. Liu , K. Adair , R. Li , L. Zhang , R. Yang , S. Lu , H. Huang , X. Sun , J. Mater. Chem. A 2018, 6, 23712.

[128] X. Xu , G. Hou , X. Nie , Q. Ai , Y. Liu , J. Feng , L. Zhang , P. Si , S. Guo , L. Ci , J. Power Sources 2018, 400, 212.

[129] X. Yang , J. Luo , X. Sun , Chem. Soc. Rev. 2020, 49, 2140. 32118221 10.1039/c9cs00635d

[130] N. Kamaya , K. Homma , Y. Yamakawa , M. Hirayama , R. Kanno , M. Yonemura , T. Kamiyama , Y. Kato , S. Hama , K. Kawamoto , A. Mitsui , Nat. Mater. 2011, 10, 682. 21804556 10.1038/nmat3066

[131] F. Chen , S. Cheng , J.‐B. Liu , S. Li , W. Ouyang , B. Liu , ACS Appl. Mater. Interfaces 2021, 13, 22438. 33881293 10.1021/acsami.1c03227

[132] C. Wang , K. R. Adair , J. Liang , X. Li , Y. Sun , X. Li , J. Wang , Q. Sun , F. Zhao , X. Lin , R. Li , H. Huang , L. Zhang , R. Yang , S. Lu , X. Sun , Adv. Funct. Mater. 2019, 29, 1900392.

[133] W. H. Meyer , Adv. Mater. 1998, 10, 439. 21647973 10.1002/(SICI)1521-4095(199804)10:6<439::AID-ADMA439>3.0.CO;2-I

[134] H. Marceau , C.‐S. Kim , A. Paolella , S. Ladouceur , M. Lagacé , M. Chaker , A. Vijh , A. Guerfi , C. M. Julien , A. Mauger , M. Armand , P. Hovington , K. Zaghib , J. Power Sources 2016, 319, 247.

[135] D.‐D. Han , S. Liu , Y.‐T. Liu , Z. Zhang , G.‐R. Li , X.‐P. Gao , J. Mater. Chem. A 2018, 6, 18627.

[136] W. Yang , W. Yang , J. Feng , Z. Ma , G. Shao , Electrochim. Acta 2016, 210, 71.

[137] M. Liu , H. R. Jiang , Y. X. Ren , D. Zhou , F. Y. Kang , T. S. Zhao , Electrochim. Acta 2016, 213, 871.

[138] P. Dhatarwal , R. J. Sengwa , J. Polym. Res. 2017, 24, 135.

[139] S. Bag , C. Zhou , P. J. Kim , V. G. Pol , V. Thangadurai , Energy Storage Mater. 2020, 24, 198.

[140] a) X. Wang , X. Hao , Z. Hengjing , X. Xia , J. Tu , Electrochim. Acta 2020, 329, 135108;

[141] X. Judez , H. Zhang , C. Li , G. G. Eshetu , Y. Zhang , J. A. González‐Marcos , M. Armand , L. M. Rodriguez‐Martinez , J. Phys. Chem. Lett. 2017, 8, 3473. 28696704 10.1021/acs.jpclett.7b01321

[142] J.‐Q. Huang , Q. Zhang , F. Wei , Energy Storage Mater. 2015, 1, 127.

[143] S.‐H. Chung , A. Manthiram , Adv. Mater. 2014, 26, 7352. 25219844 10.1002/adma.201402893

[144] H. Wang , W. Zhang , H. Liu , Z. Guo , Angew. Chem., Int. Ed. 2016, 55, 3992. 10.1002/anie.201511673 26889652

[145] S. H. Chung , A. Manthiram , J. Phys. Chem. Lett. 2014, 5, 1978. 26273884 10.1021/jz5006913

[146] B. B. Zheng , L. W. Yu , Y. Zhao , J. Y. Xi , Electrochim. Acta 2019, 295, 910.

[147] J.‐Q. Huang , Q. Zhang , H.‐J. Peng , X.‐Y. Liu , W.‐Z. Qian , F. Wei , Energy Environ. Sci. 2014, 7, 347.

[148] T. Lei , W. Chen , W. Lv , J. Huang , J. Zhu , J. Chu , C. Yan , C. Wu , Y. Yan , W. He , J. Xiong , Y. Li , C. Yan , J. B. Goodenough , X. Duan , Joule 2018, 2, 2091.

[149] Y. He , Y. Qiao , Z. Chang , X. Cao , M. Jia , P. He , H. Zhou , Angew. Chem., Int. Ed. 2019, 58, 11774. 10.1002/anie.201906055 31210379

[150] Z. Du , C. Guo , L. Wang , A. Hu , S. Jin , T. Zhang , H. Jin , Z. Qi , S. Xin , X. Kong , Y. G. Guo , H. Ji , L. J. Wan , ACS Appl. Mater. Interfaces 2017, 9, 43696. 29172433 10.1021/acsami.7b14195

[151] S. H. Chung , A. Manthiram , Adv. Funct. Mater. 2014, 24, 5299.

[152] F. Pei , L. L. Lin , A. Fu , S. G. Mo , D. H. Ou , X. L. Fang , N. F. Zheng , Joule 2018, 2, 323.

[153] D. K. Lee , Y. Chae , H. Yun , C. W. Ahn , J. W. Lee , ACS Nano 2020, 14, 9744. 32806058 10.1021/acsnano.0c01452

[154] M. Tian , F. Pei , M. S. Yao , Z. H. Fu , L. L. Lin , G. D. Wu , G. Xu , H. Kitagawa , X. L. Fang , Energy Storage Mater. 2019, 21, 14.

[155] S. Imtiaz , Z. A. Zafar , R. Razaq , D. Sun , Y. Xin , Q. Li , Z. L. Zhang , L. Zheng , Y. H. Huang , J. A. Anderson , Adv. Mater. Interfaces 2018, 5, 1800243.

[156] J.‐L. Yang , S.‐X. Zhao , Y.‐M. Lu , X.‐T. Zeng , W. Lv , G.‐Z. Cao , J. Mater. Chem. A 2020, 8, 231.

[157] H. J. Peng , Z. W. Zhang , J. Q. Huang , G. Zhang , J. Xie , W. T. Xu , J. L. Shi , X. Chen , X. B. Cheng , Q. Zhang , Adv. Mater. 2016, 28, 9551. 27629655 10.1002/adma.201603401

[158] Y. Song , S. Zhao , Y. Chen , J. Cai , J. Li , Q. Yang , J. Sun , Z. Liu , ACS Appl. Mater. Interfaces 2019, 11, 5687. 30714710 10.1021/acsami.8b22014

[159] P. Han , A. Manthiram , J. Power Sources 2017, 369, 87.

[160] L. Fan , M. Li , X. Li , W. Xiao , Z. Chen , J. Lu , Joule 2019, 3, 361.

[161] a) C. Chen , Q. B. Jiang , H. F. Xu , Y. P. Zhang , B. K. Zhang , Z. Y. Zhang , Z. Lin , S. Q. Zhang , Nano Energy 2020, 76, 105033;

[162] L.‐P. Hou , X.‐Q. Zhang , B.‐Q. Li , Q. Zhang , Mater. Today 2021, 45, 62.

[163] W. Y. Wu , J. Duan , J. Y. Wen , Y. W. Chen , X. Y. Liu , L. Q. Huang , Z. F. Wang , S. Y. Deng , Y. H. Huang , W. Luo , Sci. China Chem. 2020, 63, 1483.

[164] X.‐B. Cheng , J.‐Q. Huang , Q. Zhang , J. Electrochem. Soc. 2017, 165, A6058.

[165] E. Peled , D. Golodnitsky , G. Ardel , J. Electrochem. Soc. 1997, 144, L208.

[166] J. R. Akridge , Y. V. Mikhaylik , N. White , Solid State Ionics 2004, 175, 243.

[167] A. Rosenman , E. Markevich , G. Salitra , D. Aurbach , A. Garsuch , F. F. Chesneau , Adv. Energy Mater. 2015, 5, 1500212.

[168] D. Aurbach , E. Pollak , R. Elazari , G. Salitra , C. S. Kelley , J. Affinito , J. Electrochem. Soc. 2009, 156, A694.

[169] X. Liang , Z. Y. Wen , Y. Liu , M. F. Wu , J. Jin , H. Zhang , X. W. Wu , J. Power Sources 2011, 196, 9839.

[170] S. Xiong , K. Xie , Y. Diao , X. Hong , Electrochim. Acta 2012, 83, 78.

[171] J.‐S. Kim , D.‐J. Yoo , J. Min , R. A. Shakoor , R. Kahraman , J. W. Choi , ChemNanoMat 2015, 1, 240.

[172] W. S. Jia , C. Fan , L. P. Wang , Q. J. Wang , M. J. Zhao , A. J. Zhou , J. Z. Li , ACS Appl. Mater. Interfaces 2016, 8, 15399. 27237827 10.1021/acsami.6b03897

[173] J. Li , L. Zhang , F. Qin , B. Hong , Q. Xiang , K. Zhang , J. Fang , Y. Lai , J. Power Sources 2019, 442, 227232.

[174] W. Y. Li , H. B. Yao , K. Yan , G. Y. Zheng , Z. Liang , Y. M. Chiang , Y. Cui , Nat. Commun. 2015, 6, 7436. 26081242 10.1038/ncomms8436

[175] Z. X. Liu , S. Bertolin , P. B. Balbuena , P. P. Mukherjee , ACS Appl. Mater. Interfaces 2016, 8, 4700. 26836249 10.1021/acsami.5b11803

[176] C. Yan , X. B. Cheng , C. Z. Zhao , J. Q. Huang , S. T. Yang , Q. Zhang , J. Power Sources 2016, 327, 212.

[177] C.‐Z. Zhao , X.‐B. Cheng , R. Zhang , H.‐J. Peng , J.‐Q. Huang , R. Ran , Z.‐H. Huang , F. Wei , Q. Zhang , Energy Storage Mater. 2016, 3, 77.

[178] S. S. Zhang , Electrochim. Acta 2012, 70, 344.

[179] R. Cao , J. Chen , K. S. Han , W. Xu , D. Mei , P. Bhattacharya , M. H. Engelhard , K. T. Mueller , J. Liu , J.‐G. Zhang , Adv. Funct. Mater. 2016, 26, 3059.

[180] Z. Lin , Z. C. Liu , W. J. Fu , N. J. Dudney , C. D. Liang , Adv. Funct. Mater. 2013, 23, 1064.

[181] H.‐L. Wu , M. Shin , Y.‐M. Liu , K. A. See , A. A. Gewirth , Nano Energy 2017, 32, 50.

[182] S. Li , H. Dai , Y. Li , C. Lai , J. Wang , F. Huo , C. Wang , Energy Storage Mater. 2019, 18, 222.

[183] L. Jia , T. Wu , J. Lu , L. Ma , W. Zhu , X. Qiu , ACS Appl. Mater. Interfaces 2016, 8, 30248. 27753479 10.1021/acsami.6b10366

[184] W. Zeng , M. M.‐C. Cheng , S. K.‐Y. Ng , Electrochim. Acta 2019, 319, 511.

[185] S. Liu , G. R. Li , X. P. Gao , ACS Appl. Mater. Interfaces 2016, 8, 7783. 26981849 10.1021/acsami.5b12231

[186] W. Guo , W. Zhang , Y. Si , D. Wang , Y. Fu , A. Manthiram , Nat. Commun. 2021, 12, 3031. 34050171 10.1038/s41467-021-23155-3 PMC8163853

[187] F. Wu , J. T. Lee , N. Nitta , H. Kim , O. Borodin , G. Yushin , Adv. Mater. 2015, 27, 101. 25367318 10.1002/adma.201404194

[188] a) F. Wu , J. Qian , R. Chen , J. Lu , L. Li , H. Wu , J. Chen , T. Zhao , Y. Ye , K. Amine , ACS Appl. Mater. Interfaces 2014, 6, 15542; 25100666 10.1021/am504345s

[189] Y. Xiao , B. Han , Y. Zeng , S. S. Chi , X. Zeng , Z. Zheng , K. Xu , Y. Deng , Adv. Energy Mater. 2020, 10, 1903937.

[190] M. L. Gordin , F. Dai , S. Chen , T. Xu , J. Song , D. Tang , N. Azimi , Z. Zhang , D. Wang , ACS Appl. Mater. Interfaces 2014, 6, 8006. 24833106 10.1021/am501665s

[191] N. Azimi , W. Weng , C. Takoudis , Z. Zhang , Electrochem. Commun. 2013, 37, 96.

[192] P. Y. Chen , C. Yan , P. Chen , R. Zhang , Y. X. Yao , H. J. Peng , L. T. Yan , S. Kaskel , Q. Zhang , Angew. Chem., Int. Ed. 2021, 60, 18031. 10.1002/anie.202101958 34058049

[193] A. J. Hu , W. Chen , X. C. Du , Y. Hu , T. Y. Lei , H. B. Wang , L. X. Xue , Y. Y. Li , H. Sun , Y. C. Yan , J. P. Long , C. Z. Shu , J. Zhu , B. H. Li , X. F. Wang , J. Xiong , Energy Environ. Sci. 2021, 14, 4115.

[194] Q. Jin , X. Zhang , H. Gao , L. Li , Z. Zhang , J. Mater. Chem. A 2020, 8, 8979.

[195] Y. X. Yao , X. Q. Zhang , B. Q. Li , C. Yan , P. Y. Chen , J. Q. Huang , Q. Zhang , InfoMat 2019, 2, 379.

[196] a) A. C. Kozen , C. F. Lin , A. J. Pearse , M. A. Schroeder , X. Han , L. Hu , S. B. Lee , G. W. Rubloff , M. Noked , ACS Nano 2015, 9, 5884; 25970127 10.1021/acsnano.5b02166

[197] a) J. Zhao , L. Liao , F. Shi , T. Lei , G. Chen , A. Pei , J. Sun , K. Yan , G. Zhou , J. Xie , C. Liu , Y. Li , Z. Liang , Z. Bao , Y. Cui , J. Am. Chem. Soc. 2017, 139, 11550; 28743184 10.1021/jacs.7b05251

[198] W. Yang , W. Yang , B. Sun , S. L. Di , K. Yan , G. X. Wang , G. J. Shao , ACS Appl. Mater. Interfaces 2018, 10, 39695. 30379527 10.1021/acsami.8b14045

[199] Q. Li , F.‐L. Zeng , Y.‐P. Guan , Z.‐Q. Jin , Y.‐Q. Huang , M. Yao , W.‐K. Wang , A.‐B. Wang , Energy Storage Mater. 2018, 13, 151.

[200] J. Luo , R. C. Lee , J. T. Jin , Y. T. Weng , C. C. Fang , N. L. Wu , Chem. Commun. 2017, 53, 963. 10.1039/c6cc09248a 28044169

[201] G. Q. Ma , Z. Y. Wen , M. F. Wu , C. Shen , Q. S. Wang , J. Jin , X. W. Wu , Chem. Commun. 2014, 50, 14209. 10.1039/c4cc05535g 25285341

[202] H. Kim , J. T. Lee , D. C. Lee , M. Oschatz , W. I. Cho , S. Kaskel , G. Yushin , Electrochem. Commun. 2013, 36, 38.

[203] F. Liu , L. Wang , Z. Zhang , P. Shi , Y. Feng , Y. Yao , S. Ye , H. Wang , X. Wu , Y. Yu , Adv. Funct. Mater. 2020, 30, 1903937.

[204] G. Ma , Z. Wen , Q. Wang , C. Shen , J. Jin , X. Wu , J. Mater. Chem. A 2014, 2, 19355.

[205] Y. B. Yang , Y. X. Liu , Z. Song , Y. H. Zhou , H. Zhan , ACS Appl. Mater. Interfaces 2017, 9, 38950. 29039907 10.1021/acsami.7b10306

[206] M. S. Kim , M.‐S. Kim , V. Do , Y. R. Lim , I. W. Nah , L. A. Archer , W. I. Cho , Nano Energy 2017, 41, 573.

[207] a) X. Yu , J. Joseph , A. Manthiram , J. Mater. Chem. A 2015, 3, 15683;

[208] Z. Yu , Y. Shao , L. Ma , C. Liu , C. Gu , J. Liu , P. He , M. Li , Z. Nie , Z. Peng , Y. Shao , Adv. Mater. 2021, 34, 2106618. 10.1002/adma.202106618 34862816

[209] D. Eroglu , K. R. Zavadil , K. G. Gallagher , J. Electrochem. Soc. 2015, 162, A982.

